# Diets, dominance hierarchies, and kleptoparasitism drive asymmetrical interactions between wolves and cougars

**DOI:** 10.1073/pnas.2511397123

**Published:** 2026-01-26

**Authors:** Wesley Binder, Joel S. Ruprecht, Jack Rabe, Matthew C. Metz, Rebecca Hutchinson, Daniel R. Stahler, Taal Levi

**Affiliations:** ^a^Department of Fisheries, Wildlife and Conservation Science, Oregon State University, Corvallis, OR 97331; ^b^Yellowstone Center for Resources, Yellowstone National Park, Mammoth Hot Springs, WY 82190; ^c^Department of Fisheries, Wildlife and Conservation Biology, University of Minnesota, St. Paul, MN 55108; ^d^Department of Electrical Engineering and Computer Science, Oregon State University, Corvallis, OR 97331

**Keywords:** competition, carnivore interactions, movement ecology, random forests, Yellowstone

## Abstract

After widespread extirpation, conservation efforts have restored large carnivores to portions of their former range. Substantial research has since focused on their ability to alter ecological communities through effects on herbivores and mesocarnivores, but the principles governing competition among multiple apex carnivores remain unclear. While mesocarnivore scavenging of apex carnivore kills drive “enemies with benefits” dynamics, subordinate apex carnivores seldom rely on scavenging. Instead, they proficiently hunt prey themselves that dominant apex carnivores can steal, fundamentally altering the dynamics of their interactions. We used 9 y of contemporaneous GPS telemetry and 3,929 potential kill site investigations from a reconstituted community of sympatric wolves and cougars in Yellowstone National Park to test whether such apex–apex dynamics instead follow an “enemies without benefits” framework. Wolf movement and resource selection were strongly linked to cougar kills, resulting in carcass theft (i.e., kleptoparasitism) that drove their interactions and led to cougar mortalities. In contrast, cougars avoided wolf kills, were tied to escape terrain, and did not kill wolves, producing an asymmetric dynamic that persisted across distinct seasonal contexts. However, as elk (the shared primary prey) declined longitudinally, cougars shifted their diets to smaller-bodied deer that were kleptoparasitized by wolves at one-sixth the rate of elk kills. Our findings demonstrate that prey diversity and landscape structure are critical for subordinate apex carnivores who experience severe interference competition from their dominant counterparts. This dynamic extends trophic theory and provides a framework for predicting the coexistence outcomes of carnivore restoration and the ongoing recolonization of wolves.

## Significance

Theory of carnivore interactions has focused on apex carnivores suppressing and facilitating mesocarnivores through “enemies with benefits” dynamics, but the mechanisms governing apex–apex interactions remain unclear. Using 9 y of GPS and predation data from Yellowstone wolves and cougars, we show that wolf theft of cougar kills drives their interactions, leads to cougar mortalities, and is not reciprocal, creating an “enemies without benefits” dynamic. However, following a decline in elk, cougars shifted their diets to smaller-bodied deer that reduce handling times and thus the risk of wolf encounters. Our findings demonstrate that landscape structure and prey diversity, not just prey abundance, determine apex carnivore coexistence, providing a predictive framework for carnivore restoration globally.

After two centuries of widespread declines in the abundance and distribution of large mammalian carnivores, populations in many areas are now recovering (1–3). Large carnivore recoveries have led to substantial research and debate concerning their impact on ecological communities, including whether large carnivores suppress large herbivore populations and thus indirectly benefit plant communities, a phenomenon called trophic cascades (3). Similar interactions can occur within carnivore communities, where apex carnivore suppression of mesocarnivores can lead to increases in the abundance of their prey or smaller carnivore competitors (4, 5). Apex carnivores account for an average of 33% of mesocarnivore mortalities across systems, but they partially offset these costs by provisioning carrion subsidies that facilitate mesocarnivores, leading to risk–reward tradeoffs that have been referred to as “enemies with benefits” (6, 7). Comparable dynamics can arise among apex carnivores, particularly when competitive hierarchies are ambiguous. For example, African lions (*Panthera leo*) and spotted hyenas (*Crocuta crocuta*) each steal (i.e., kleptoparasitism) and scavenge each other’s kills with dominance at kill sites determined by body and group size (8). However, many apex–apex interactions involve clear dominance hierarchies similar to apex–mesocarnivore interactions but fundamentally differ because subordinate apex carnivores are proficient at hunting large prey themselves and thus do not rely on scavenging opportunities (9, 10). While this behavior would seemingly reduce interactions, instead the focal point of encounters could simply shift to the kill sites of the subordinate carnivore if kleptoparasitism by the dominant carnivore is prevalent. These asymmetric interactions between apex carnivores would lead to an “enemies without benefits” dynamic that has the potential to inhibit carnivore coexistence and restoration efforts, yet the underlying mechanisms of such interactions are poorly understood.

Coexistence among apex carnivores can be modulated by landscape structure and the diversity and abundance of prey. For example, leopards (*Panthera pardus*) mitigate kleptoparasitism by caching prey in trees, allowing these subordinate felids to occupy prey-rich areas with suitable arboreal caching sites (11, 12). In contrast, cheetahs (*Acinonyx jubatus*) and wild dogs (*Lycaon pictus*) suffer from high rates of kleptoparasitism and intraguild mortalities that can exclude them from otherwise suitable habitat when abundant prey lead to high densities of lions and spotted hyenas (9, 10). These subordinate carnivores can benefit indirectly from reduced prey density due to a relaxation in interference competition and compensate with high hunting efficiency (9, 10). Examples from Africa highlight that interference competition can substantially influence the fitness and distribution of subordinate carnivores depending on the context of landscape structure and prey availability. However, critical questions remain, particularly surrounding the nature of interactions among apex carnivores in temperate regions, many of which are recently reconstituted and have much lower prey diversity. Understanding how competitive dominance, landscape structure, and prey communities mediate carnivore interactions is critical for these ongoing recovery efforts.

Recolonizing wolf (*Canis lupus*) and cougar (*Puma concolor*) populations in western North America provide an ideal system to extend theoretical frameworks for apex carnivore interactions. Both species were extensively persecuted and extirpated for much of the 20th century before government protections and reintroductions spurred ongoing recolonizations (3, 13, 14). These species are once again sympatric, and their reestablished competitive dynamics have now been monitored across variable ecological conditions for three decades (14, 15). Although wolves and cougars are similar in size—with male cougars typically the largest—group-living wolves are competitively dominant over solitary cougars (15). Cougars can avoid agonistic encounters by climbing trees or navigating rugged terrain, but such habitat selection is less effective at mitigating kleptoparasitism (15). If kleptoparasitism is a powerful force driving competitive dynamics among wolves and cougars, we would expect it to drive their movements, interactions, and intraguild mortalities. We would further expect the strength of this interference competition to vary in space and time. On longer time scales or across spatial contexts, changes in prey abundance and community composition could alter the severity of interference competition. For example, large-bodied prey such as elk (*Cervus canadensis*) require extended handling times compared to smaller prey like deer (*Odocoileus spp.*), increasing the temporal window for kleptoparasitic discovery (15, 16). The strength of interference competition could also vary across seasons. Prey biomass acquisition rates for wolves are lowest in summer, which could motivate increased rates of kleptoparasitism. However, less wolf pack cohesion during summer could reduce their competitive dominance, and the presence of bears that have already usurped carcasses could further diminish opportunities for wolves to kleptoparasitize cougar kills (14, 15). Accordingly, understanding the mechanisms that modify the intensity of such enemies without benefits interactions among wolves and cougars could highlight ecological conditions that support apex carnivore coexistence.

Here, we use 9 y of contemporaneous GPS telemetry and comprehensive predation monitoring from northern Yellowstone National Park to test if wolf–cougar interactions follow enemies without benefits dynamics and identify the mechanisms enabling subordinate carnivore coexistence. Specifically, we test if wolf kleptoparasitism of cougar kills drives their interactions across seasonal contexts, leads to intraguild killing, and is mediated by the prey size of cougars. We further test if cougars actively avoid wolf kills or modify their space use in response to wolves. We accomplish this by documenting interspecific killing through mortality investigations of monitored individuals. We recorded wolf and cougar kill sites and prey use through field investigations at clusters of GPS fixes. We then used these data to train separate machine learning models to predict kills in summer and winter, allowing us to pair probable kill sites with all GPS movements. Contemporaneous GPS data were then used to identify whether wolf and cougar interactions were kleptoparasitic (occurred at predicted kill sites while competitors were present). We quantified attraction to, and encounters with, both individuals and predicted kill sites from the competitor species. Finally, we exploited a substantial decline in elk density over the last 25 y to test whether dietary shifts toward smaller-bodied deer reduced kleptoparasitism rates. We hypothesized that prey body size directly links dietary composition to the intensity of interference competition in apex carnivore hierarchies, providing a mechanism for subordinate carnivore persistence. By integrating movement ecology, predation patterns, machine learning-based kill site identification, and long-term dietary data spanning changing prey availability, we provide a framework for understanding competition in reconstituted apex carnivore communities to inform the ongoing recovery of these species in North America, as well as carnivore restoration efforts worldwide.

## Materials and Methods

### Study Area.

**Fig. 1.**
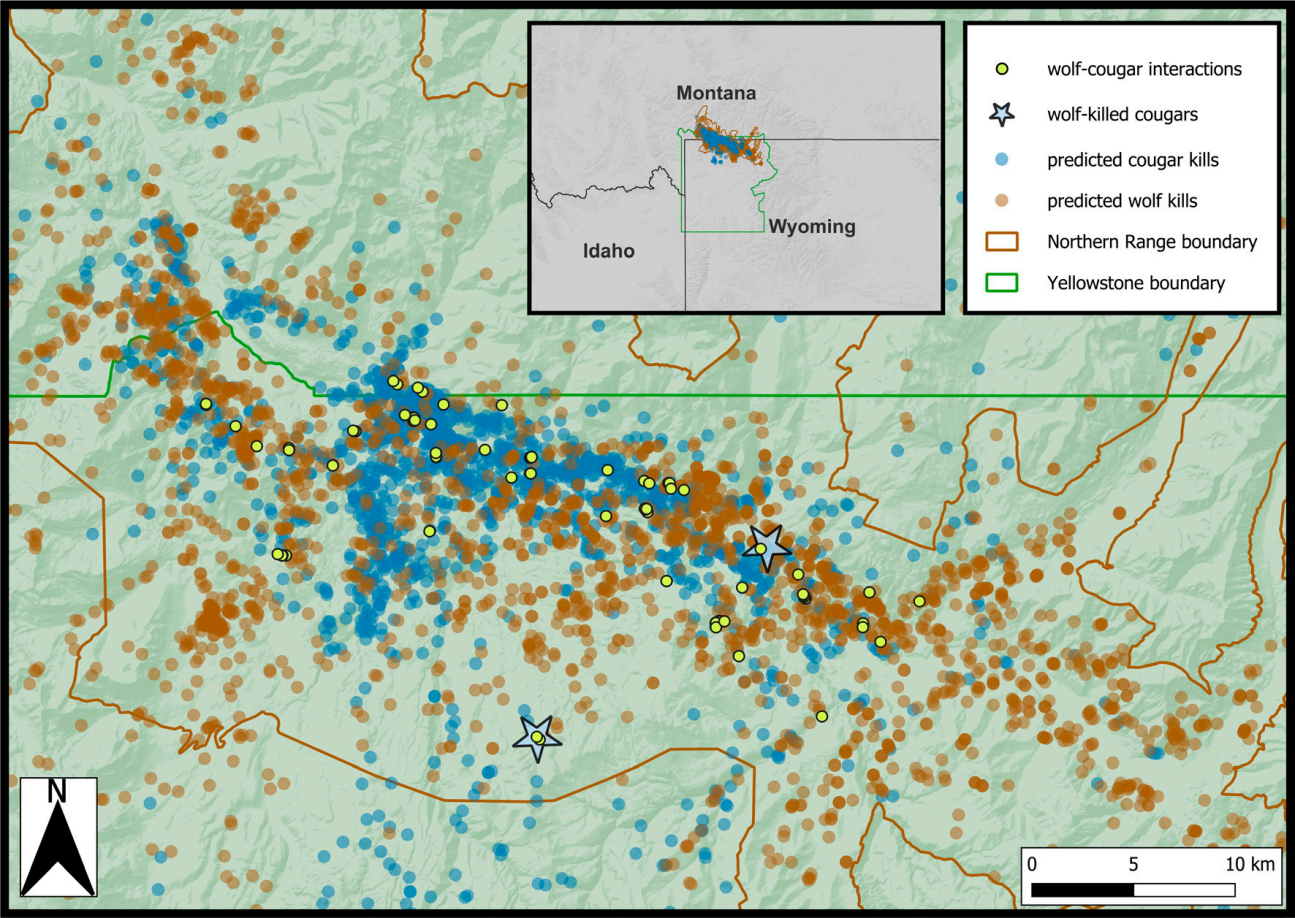
Map of northern Yellowstone National Park that includes wolf–cougar interactions (individuals <250 m at the same time), predicted kill sites from the random forests models, and known cougar mortalities attributed to wolves from 2016 to 2024.

Our study was conducted in northern Yellowstone National Park and the adjacent lands north of the park boundary (Fig. 1). Cougars naturally recolonized this system during the latter part of the 20th century, while wolves were reintroduced here in 1995 (14, 15). Now, this area hosts a diverse multicarnivore (e.g., four species of large carnivores), multiprey (e.g., eight species of ungulates), and multiscavenger (including mesocarnivore and avian species) system (14). Elevations range from 1,500 to 2,900 m and are influenced by the prominent Yellowstone River and its tributaries (15). These topographically complex riparian areas fragment the otherwise relatively open landscape. Winter snow accumulations vary considerably both within the study area and between years, with more snow accumulating in the eastern areas that contain higher elevations (15).

### Data Collection.

Wolves and cougars were captured and fit with Global Positioning System (GPS) telemetry collars (Vectronic Aerospace or Telonics) in northern Yellowstone National Park using helicopters and trained hound dogs, respectively. Data used here were collected from February 2016 through March 2024 (hereafter “2016 to 2024”). Data were restricted to winter (November–December, Feb–March) and spring-summer (May–July) months when predation monitoring occurred. Prey species of wolves and cougars were limited to ungulates for all analyses. Additional information regarding GPS collar programming and data filtering can be found in *SI Appendix*, *A*, Text S1.

### Predation Monitoring.

**Fig. 2.**
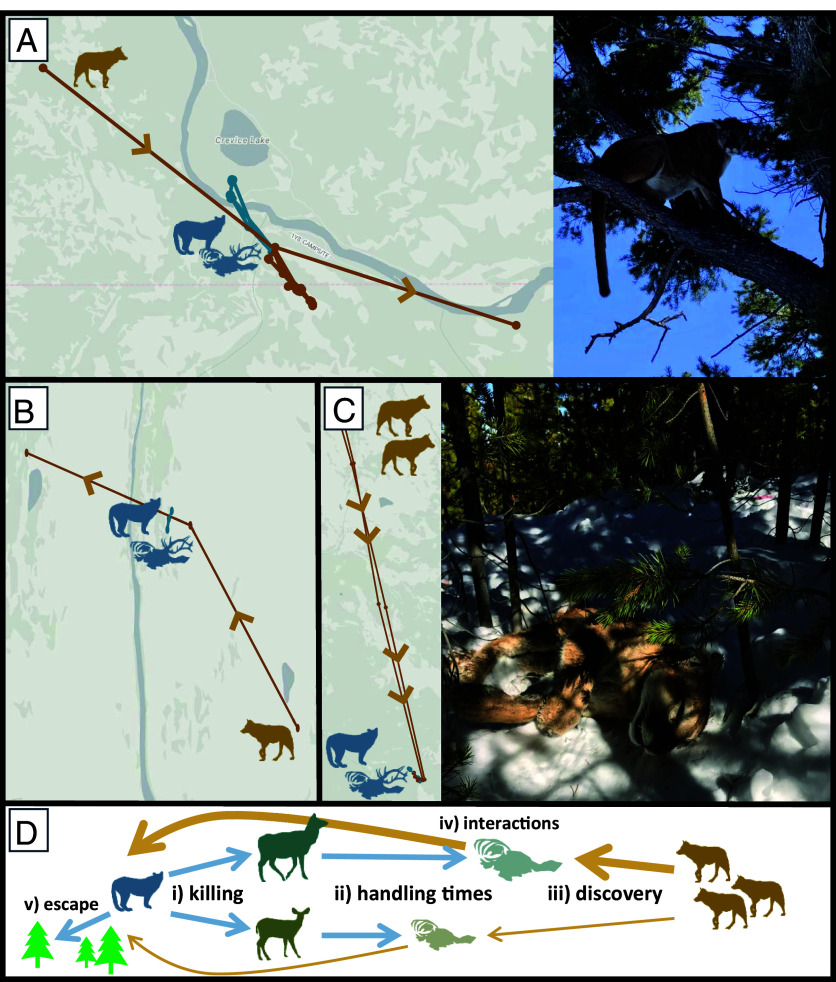
GPS fixes associated with wolf (brown points/lines) kleptoparasitism of cougar (blue points/lines) kills. (*A*) Wolf 1273M found cougar M227’s cow elk kill, and these individuals took turns feeding on the carcass throughout the day. Other wolves were likely present, but large trees and rugged terrain were available to offer refuge for M227 (photo: J. Rabe/NPS). (*B*) Wolf 1273M and the Rescue Creek pack stole and quickly consumed F223’s cow elk kill as this female cougar and her kittens moved to safe terrain nearby. (*C*) Wolves from the Eight Mile Creek pack killed cougar F207 when they discovered her on a bull elk kill. We speculated that the regenerating lodgepole pine trees in this area were too short/thin for a cougar to climb and escape wolves (photo: D. Stahler/NPS). (*D*) We hypothesized that wolf kleptoparasitism of cougar kills would drive their interactions, occur more frequently when cougars killed larger-bodied elk compared to deer (due to longer handling times), and result in cougar mortalities if no “escape” terrain was available. Bold arrows represent the increased likelihood of wolves detecting a kill and subsequently interacting with a cougar. Carcass phylopic provided by K. Cassidy.

We documented intraguild mortalities by conducting field necropsies of monitored individuals. We assigned cause-specific mortalities when we were able to examine the deceased individuals within 2 to 3 d of their death or had clear evidence of how they died (e.g., bite wounds, internal hemorrhaging, GPS data, etc.; Fig. 2*C*).

Wolf and cougar diets were quantified by searching aggregations of GPS fixes (hereafter, “clusters”), as well as through ground- and aerial-based observations (*SI Appendix, A*, Text S2). Prey remains were assigned as either carnivore kills or scavenging events based on site disturbance and carcass attributes (e.g., time since death, entry points, etc.) (17). To assess changes in carnivore diets in response to a decline in the elk population, we compared these data to estimates of wolf and cougar diets from 1998 to 2005 when elk densities were high (14, 15). We used binomial generalized linear models (GLMs) to evaluate changes to the odds of prey use for each carnivore and Pianka’s overlap index to assess changes to their dietary overlap (18, 19). Although mule deer (*Odocoileus hemionus*) occur at much higher densities (15), we combined mule and white-tailed deer (*Odocoileus virginianus*) for all analyses due to the high number of unknown deer species within diets.

### Predictive Kill Models.

As we could not visit all clusters from the collared wolves and cougars used in our movement analyses, we trained random forest models on ground-truthed GPS cluster searches for wolves and cougars from 2016 to 2022 to predict whether clusters contained a feeding event (ungulate kill or scavenging event; hereafter “kills”) (20). This was done to ensure competitor movements used in our movement analyses were associated with probable kill sites such that selection for the competitor and competitor’s kill site could be distinguished. We estimated the predictive performance of random forest models with subsets of ground-truthed clusters that were not used in model training. Kill predictions were binary and did not differentiate between ungulate species. Predicted kill sites were derived by generating clusters for wolf and cougar GPS data and applying the random forest models to classify them into kills and nonkills. The predicted kill sites were then supplied to our subsequent movement analyses. Additional information on cluster generation and the development of random forests models can be found in *SI Appendix*, *B*.

### Integrated Step-Selection Functions.

We fit integrated step-selection functions (iSSFs) to wolf and cougar GPS data to estimate how their movements were influenced by environmental variables, competitors, and kill sites from the competitor species (*SI Appendix*, *A*, Text S3) (21). This framework allows for observed movements between GPS fixes (i.e., “steps”) to be compared with hypothetical movements to make inference on the set of factors influencing movement decisions. For each observed step, we generated twenty random steps originating from the same initial location that represent plausible movements that the animal could have made but did not. Random steps were drawn from individual-specific distributions describing step lengths (i.e., the Euclidean distance between successive GPS relocations) and turning angles (i.e., the angle describing the change in direction from the previous step). We sampled from a gamma distribution fit to the empirical distribution of step lengths and a von Mises distribution fit to the empirical distribution of turning angles for each individual to generate random steps (21). We included the movement attributes of each step (i.e., the natural logarithm of the step length and the cosine of the turning angle) as parameters in each model because failing to account for the movement process can bias inference on resource selection (22). Only GPS data recorded at 1-h fix rates were included to ensure contemporaneous GPS data were available from the competitor species for each movement, thus reducing the number of missed interactions. We considered a competitor’s kill “available” for the focal individual to interact with for 30 d after the kill was made and assumed that beyond this time it would no longer have the potential to appreciably influence movements or behavior (7). We primarily make inference from a combined model pooling winter and summer movements, but we also ran season-specific models to evaluate seasonal differences.

Before assessing the role of interspecific interactions on individual carnivore movements, we first fit models containing only landscape and movement variables (hereafter “habitat”). Environmental covariates used for our analyses included topographic roughness, percent tree cover, and snow depth (*SI Appendix*, *A*, Text S3) (23) as previous work has demonstrated their strong influence on both wolf and cougar movements (14, 15, 24). For each species, we quantified the relative probability of selection as follows (21):

“Habitat” model:

[1] $$\begin{array}{c} \text{w}(\text{x})=\exp({\text{β}}_{\text{step}\;\text{length}}\times \ln\left(\text{step}\;\text{length}\right)+{\text{β}}_{\text{turning}\;\text{angle}}\times \cos\left(\text{turning}\;\text{angle}\right)\\ +{\text{β}}_{\text{topographic}\;\text{roughness}}\times \text{topographic}\;\text{roughness}+{\text{β}}_{\text{forest}\;\text{cover}}\times \text{forest}\;\text{cover}\\ +{\text{β}}_{\text{snow}\;\text{depth}}\times \text{snow}\;\text{depth}). \end{array}$$

We then nested the habitat model within a series of models to quantify selection for either proximity (attraction models) or encounters (encounter models) with heterospecific competitors and their kills.

**Fig. 3.**
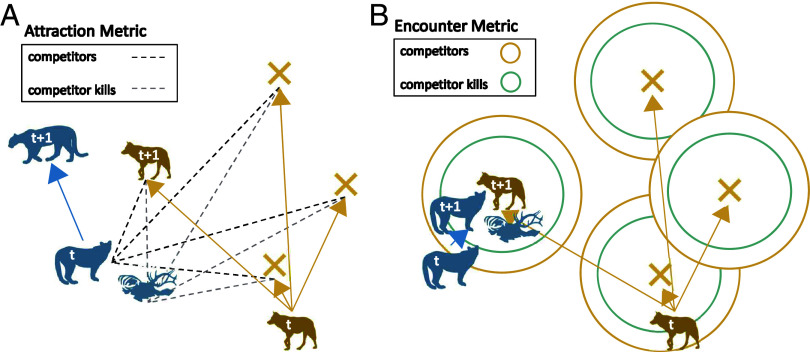
Illustrations of the metrics used to quantify interactions between wolves, cougars, and their kills. We used integrated step-selection functions that compare “observed” movements (wolves at “t + 1”) to available movements (“**×**”s; n = 20 in analyses, with three movements displayed here). (*A*) First, the attraction metric (Eq. **2**) where the natural logarithm of the Euclidean distance was measured between the focal individual and the nearest competitor/competitor kill. To avoid the competitor’s behavior confounding this metric, distance was measured between the focal individual at the end of the step (t + 1) and the competitor at the beginning of the step (t; Eq. **2**). (*B*) Second, the binomial encounter metric (Eqs. **3** and **4**) where interactions occurred if a competitor was within 250 m (at the same time) and/or a competitor kill was within 100 m of the focal individual (Eq. **3**). Panel (*B*) provides an example of the competitor being present at its kill site, indicating that kleptoparasitism likely occurred (represented in the “encounter: competitor present” model; Eq. **4**).

The attraction models used contemporaneous movement data to quantify the degree to which individuals move toward or away from competitors and their kills. We added an “attraction” metric to the habitat model using the natural logarithm of the Euclidean distance to the nearest individual competitor and to the nearest competitor’s kill (using the predicted kill sites from the random forest models; Fig. 3*A*). Because the movements of a competitor during an interaction might confound inferences when using this metric (e.g., fleeing the area), we measured the distance between the focal individual at the end of the step to the competitor at the beginning of the step. Our attraction model took the form:

“Attraction” model:

[2] $$\begin{array}{c} \text{w}(\text{x})=\text{habitat}\;\text{model}\times \exp({\text{β}}_{\text{competitors}}\times \ln\left(\text{distance}\;\text{to}\;\text{competitor}\right)\\ +{\text{β}}_{\text{competitor}\;\text{kills}}\times \ln\left(\text{distance}\;\text{to}\;\text{competitor}\;\text{kill}\right)) \end{array}$$

To determine whether cougars modify habitat selection in response to wolf proximity as a potential behavioral mechanism to reduce risk, we additionally evaluated whether their selection for topographic roughness and canopy cover varied as a function of distance to the nearest wolf using interaction terms:

“Attraction: habitat interaction” model:

[3] $$\begin{array}{c} \text{w}(\text{x})=\text{habitat}\;\text{model}\times \exp({\text{β}}_{\text{competitors}}\times \ln\left(\text{distance}\;\text{to}\;\text{competitor}\right)\\ +{\text{β}}_{\text{competitorsXtopo}}\times (\left(\ln\left(\text{distance}\;\text{to}\;\text{competitor}\right)\times \text{topographic}\;\text{roughness}\right)\\ +{\text{β}}_{\text{competitorsXcover}}\times \left(\ln\left(\text{distance}\;\text{to}\;\text{competitor}\right)\times \text{forest}\;\text{cover}\right)). \end{array}$$

The outcome of attraction and avoidance behaviors can ultimately lead to interactions in space between competitors or their kills. To determine whether wolves and cougars encountered the other species or their kills more often than expected by chance, we replaced the attraction metrics with binary classifications for interactions where we considered an encounter with a competitor or a competitor’s kill to have occurred if the endpoint of a focal individual’s movement was within 250 m or 100 m, respectively (Fig. 3*B*). As these distances are short relative to studies that have conducted similar analyses (25), we assumed them to be conservative estimates of the number of encounters that actually occurred and thus minimized type I (false-positive) errors (*SI Appendix*, *A*, Table S1 for a sensitivity analysis). Encounters were modeled as follows:

“Encounter” model:

[4] $$\begin{array}{c} \text{w}(\text{x})=\text{habitat}\;\text{model}\times \exp({\text{β}}_{\text{competitors}}\times \text{competitor}\;\text{encounter}\\ +{\text{β}}_{\text{competitor}\;\text{kills}}\times \text{competitor}\;\text{kill}\;\text{encounter}) \end{array}$$

We created an additional model that included an interaction term between “competitor encounter” and “competitor’s kill encounter” to capture whether selection for kill sites was higher when the competitor was still present (indicative of selection for kleptoparasitism) or when the competitor was absent (indicative of selection for scavenging).

“Encounter: competitor present” model:

[5] $$\begin{array}{c} \text{w}(\text{x})=\text{habitat}\;\text{model}\times \exp({\text{β}}_{\text{competitors}}\times \text{competitor}\;\text{encounter}\\ +{\text{β}}_{\text{competitor}\;\text{kills}}\times \text{competitor}\;\text{kill}\;\text{encounter}+{\text{β}}_{\text{competitor}\;\text{present}}\\ \times \left(\text{competitor}\;\text{encounter}\times \text{competitor}\;\text{kill}\;\text{encounter}\right)) \end{array}$$

**Fig. 4.**
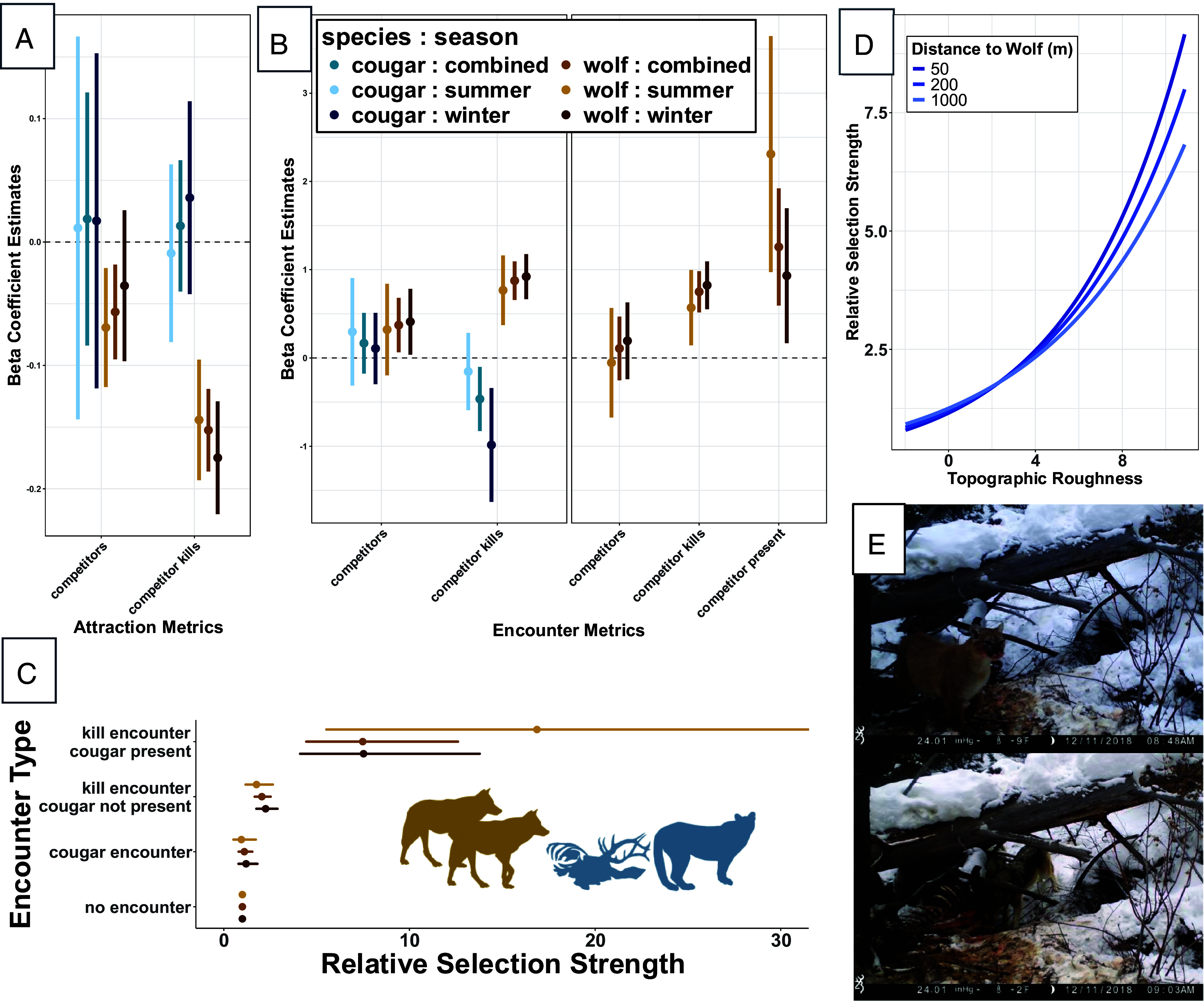
Results from the wolf and cougar movement models. Beta coefficient estimates from the integrated step-selection functions are shown to compare the (*A*) attraction (Eq. **2**) and (*B*) encounter (Eqs. **4** and **5**) metrics used to assess the drivers of wolf–cougar interactions. For the attraction metrics, estimates below the dashed line represent attraction (i.e., selection for “less” distance), while estimates for the encounter metrics are above the dashed line when encounters are selected for more often than expected by chance. (*C*) Wolf relative selection strength for the encounter metrics where “no encounter” is the reference category by which the likelihoods of selection for a cougar encounter, cougar kill encounter (cougar not present), and cougar kill encounter (cougar present/kleptoparasitism) are estimated. The summer “kill encounter, cougar present” upper CI extends to 51.5 due to a smaller sample size of interactions. (*D*) Cougar relative selection strength for topographic roughness as proximity to the nearest wolf varies (Eq. **3**). (*E*) Trail camera images from a cougar kill that was kleptoparasitized by a pack of wolves.

We compared models for each species using Akaike’s Information Criterion (AIC) to distinguish whether more complex models including species interactions better predicted animal movement (26). We also used a relative selection strength approach that compares the relative probabilities of selection using iSSF parameters to interpret model results (Fig. 4 *C* and *D*) (27).

### Interactions at Kill Sites.

We evaluated the role of kill sites in driving wolf–cougar interactions (i.e., kleptoparasitism) by estimating the proportion of interactions that occurred at predicted kill sites. We quantified this proportion by dividing the number of times a focal individual simultaneously encountered a competitor (within 250 m) and their predicted kill (within 100 m) by the total number of competitor encounters.

We further examined wolf interactions with cougars and cougar kills of known ungulate species from field investigations to evaluate how changes to cougar diets from 1998 to 2005 to 2016 to 2024 could be influencing such relationships. These research “phases” captured significant differences in elk availability, as their densities were high (mean = 7.95 elk/km^2^) from 1998 to 2005 and much lower from 2016 to 2024 (mean = 1.9 elk/km^2^; *SI Appendix*, *A*, Text S4) (28). We estimated which cougar kills were available to wolves by ensuring they fell within the home range of at least one GPS-collared wolf, then calculated the proportions, by prey species, where a GPS-collared wolf logged at least one GPS fix within 100 m of the kill site (*SI Appendix*, *A*, Text S5). We used binomial GLMs to test if wolves were 1) more likely to find cougar-killed elk or deer carcasses, or 2) more likely to encounter a cougar at a cougar-killed elk or deer carcass (i.e., kleptoparasitic interference competition).

## Results

### Predation Monitoring.

Of the twelve known-fate mortalities of adult cougars from 2016 to 2024, two (16.7%) were definitively attributed to intraguild mortalities by wolves. In both of these events, no escape terrain (e.g., a climbable tree) was available and wolves did not consume the cougars, rather they kleptoparasitized the cougar’s elk kill (Fig. 2*C*). Four cougars were legally harvested by humans outside of Yellowstone, four were assigned as natural mortalities (e.g., malnutrition, intraspecific, etc.), and two were unknown. Ninety wolf mortalities were documented during this time, none of which were attributed to cougars. Forty-five were human-related (e.g., legal harvests, poaching, vehicle collisions, etc.), 38 were natural, and seven were unknown.

**Fig. 5.**
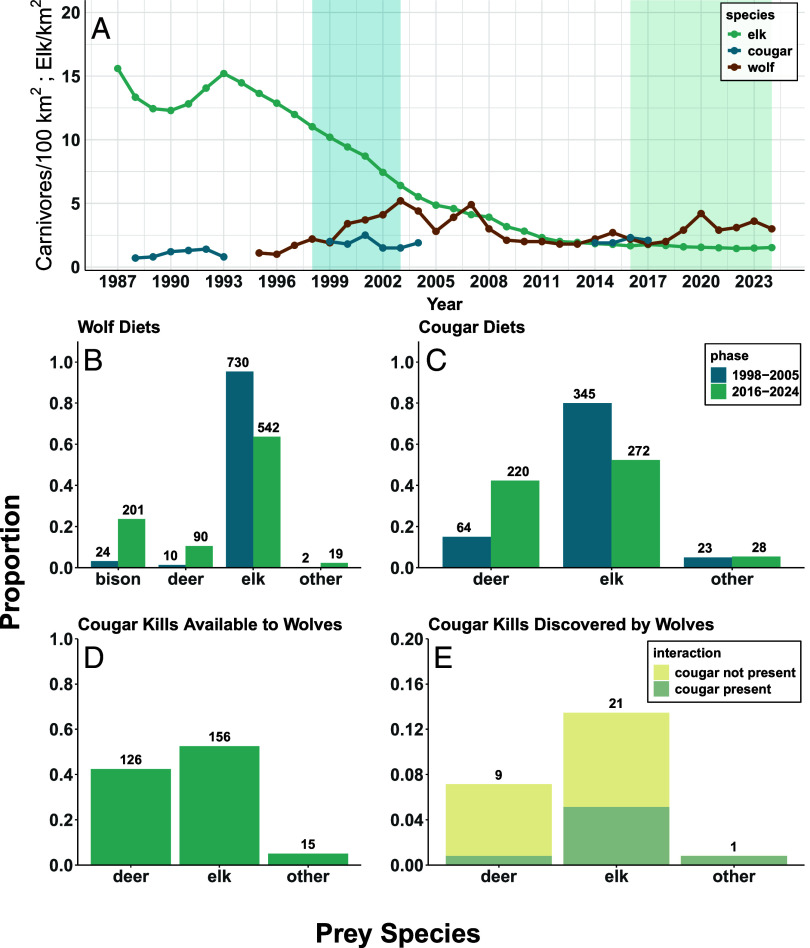
A progression of results that demonstrate how the decrease in elk, the once primary prey species of wolves and cougars, is likely reducing their interference competition in northern Yellowstone. Panel (*A*) shows density estimates of these three species. With less elk, (*B*) wolves have increased the proportion of bison and deer species in their diets, while (*C*) cougars have increased the proportion of deer in their diets (sample sizes are provided above the bars). (*D*) Although similar numbers of cougar-killed elk and deer carcasses were available to the GPS-collared wolves, (*E*) elk carcasses were discovered by wolves more often, and cougars were still present during 38% of these interactions, compared to just 11% of the wolf interactions at deer carcasses.

We conducted 3,929 potential kill site investigations and documented 852 wolf and 520 cougar feeding events on ungulate carcasses (Fig. 5 *B* and *C*). Of the wolf feeding events, 716 were determined to be wolf kills, while 136 were scavenging or kleptoparasitism. Prey species included 201 bison, 90 deer, 542 elk, and 19 carcasses of other ungulate species. Cougar feeding events included 513 that were assigned as cougar kills and 7 that were assigned as scavenging. This included 220 deer, 272 elk, and 28 carcasses of other ungulate species.

We found significant changes to wolf and cougar prey use from 1998 to 2005 to 2016 to 2024 (*SI Appendix*, Table S2 and *A*). In wolf diets, bison increased from 3.1 to 23.6%, deer increased from 1.3 to 10.6%, elk declined from 95.3 to 63.6%, and other ungulates slightly increased from 0.3 to 2.2%. Cougars reduced elk use from 79.9 to 52.3% between these periods, while deer increased from 14.8 to 42.3%, and other ungulates remained similar (5.3 to 5.4%). Wolves and cougars had very high dietary niche overlap from 1998 to 2005 (98.4%) that was reduced substantially from 2016 to 2024 (81.8%).

### Predictive Kill Models.

We trained season-specific predictive wolf kill models using 997 and 332 ground-truthed winter and summer clusters, respectively, from a total of 16 GPS-collared wolves. One hundred ninety-seven winter clusters and 104 summer clusters were verified ungulate kills. The area under the receiver operating curve for predicting kill sites on out-of-sample testing data (i.e., clusters that were not used to train the model) was 0.85 for winter and 0.84 for summer, indicating strong predictive performance (*SI Appendix*, *B*). Season-specific predictive cougar kill models were trained using 352 and 407 ground-truthed winter and summer clusters, respectively, from a total of 15 GPS-collared cougars. One hundred winter clusters and 106 summer clusters were verified ungulate kills. The area under the receiver operating curve for predicting kill sites on out-of-sample testing data was 0.94 for winter and 0.89 for summer, indicating very strong predictive performance (*SI Appendix*, *B*). After running the applicable GPS data (pertaining to the iSSF analyses) through the clustering algorithms and random forests models, a total of 3,341 wolf kills and 1,710 cougar kills were predicted across seasons (Fig. 1).

### Integrated Step-Selection Functions.

Wolves (n = 38 individuals) and cougars (n = 18 individuals) responded to each other distinctly in space, and these results were consistent across seasons (Fig. 4; combined seasonal results are reported here; see *SI Appendix*, Tables S3–S5 and *A* for all combined, winter, and summer parameter estimates). For wolves, we found the most support for the attraction model, as wolves selected for distances closer to the nearest cougar kill (β_competitor kills_ = −0.15, *P* < 0.001) which is indicative of strong attraction, as well as attraction toward the nearest cougar (β_competitors_ = −0.06, *P* = 0.003; Fig. 4*A*, Table 1, and *SI Appendix*, Fig. S1). The “encounter: competitor present” model (ΔAIC = 38.91) had substantially more support than the encounter model (ΔAIC = 51.40). Wolves selected strongly for cougar kill encounters (β_competitor kills_ = 0.75, *P* < 0.001) and selection increased further when a cougar was still present (β_competitor present_ = 1.26, *P* < 0.001). Accounting for cougar presence at kill sites with the “competitor present” covariate diminished the effect of wolf selection for cougars that was captured in the encounter model, indicating that kleptoparasitism of cougar kills disproportionately drove wolf–cougar encounters (Fig. 4 *B* and *C*, Table 1, and see *SI Appendix*, Table S1 and *A* for “encounter: competitor present” results that vary by encounter distance thresholds). The landscape variables that we controlled for had consistent effects across model structures, yet the “habitat” wolf model had poor support relative to interactive models (ΔAIC = 126.36), indicating that wolf movements were strongly influenced by cougars and their kills. As expected, wolves selected for flatter areas (β_topographic roughness_ = −0.12, *P* < 0.001) with less forest cover (β_forest cover_ = −0.06, *P* < 0.001) and snow depth (β_snow depth_ = −0.26, *P* < 0.001; Table 1).

**Table 1.** Wolf and cougar parameter estimates from each iSSF model using the combined data (winter and summer; arranged by species and ΔAIC)

Carnivore	Model	Covariate	β	SE	*P*
wolf	habitat + attraction	competitors	−0.06	0.02	0.003
	ΔAIC = 0	competitor kill	−0.15	0.02	<0.001
	habitat + “encounters:	competitors	0.11	0.17	0.534
	competitor present”	competitor kill	0.75	0.11	<0.001
	ΔAIC = 38.91	competitor present	1.26	0.33	<0.001
	habitat + encounters	competitors	0.37	0.15	0.011
	ΔAIC = 51.40	competitor kill	0.88	0.10	<0.001
	habitat only	topographic roughness	−0.12	0.01	<0.001
	ΔAIC = 126.36	forest cover	−0.06	0.01	<0.001
		snow depth	−0.26	0.02	<0.001
cougar	habitat + encounters	competitors	0.17	0.17	0.311
	ΔAIC = 0	competitor kill	−0.47	0.17	0.008
	habitat + “attraction:	competitors	0.03	0.05	0.596
	habitat interaction”	competitors × topo	−0.01	0.01	0.036
	ΔAIC = 1.45	competitors × cover	0.02	0.01	0.026
	habitat only	topographic roughness	0.13	0.01	<0.001
	ΔAIC = 4.70	forest cover	0.17	0.01	<0.001
		snow depth	−0.18	0.03	<0.001
	habitat + attraction	competitors	0.02	0.05	0.717
	ΔAIC = 8.24	competitor kill	0.01	0.03	0.619

The same environmental covariates were included in each model but are only provided for the habitat models here because estimates were consistent throughout. The parameter estimates for the attraction covariates are negative when attraction is exhibited and positive for avoidance. All other beta coefficient estimates are positive when selection is exhibited. See Fig. 3 for illustrations of the different model types and *SI Appendix*, Tables S1–S3 and *A* for all parameter estimates and season-specific results.

Seasonal wolf movements showed minor differences. There was no effect of wolf attraction to the nearest cougar in winter (β_competitor_ = −0.04, *P* = 0.244), and there was no selection for cougar encounters in the summer using the encounter model that did not account for cougar presence at kill sites (β_competitor_ = 0.32, *P* = 0.206). Strong selection for kleptoparasitic encounters was consistent across seasons, with wolves exhibiting marginally stronger selection in the summer (β_competitors_ = −0.05, *P* = 0.860; β_competitor kills_ = 0.57, *P* = 0.006; β_competitor present_ = 2.31, *P* < 0.001) compared to winter (β_competitors_ = 0.19, *P* = 0.357; β_competitor kills_ = 0.82, *P* < 0.001; β_competitor present_ = 0.93, *P* = 0.014; Fig. 4 *B* and *C*).

The combined winter and summer cougar movements showed the most support for the encounter model which was driven by avoidance of wolf kills (β_competitor kills_ = −0.47, *P* = 0.008; Fig. 4*B* and Table 1). We found similar support for the “attraction: habitat interaction” model (ΔAIC = 1.45) as cougars increased their selection for topographic roughness (β_competitorsXtopo_ = −0.01, *P* = 0.036) and decreased their selection for forest cover (β_competitorsXcover_ = 0.02, *P* = 0.026) with decreasing distance to the nearest wolf. There was no evidence that cougars otherwise avoided wolves as the attraction cougar model exhibited relatively poor support (ΔAIC = 8.24) and neither distance to wolves (β_competitors_ = 0.02, *P* = 0.717) nor their kills (β_competitor kills_ = 0.01, *P* = 0.619) helped to explain their movements (Fig. 4*A* and Table 1). Landscape variables had consistent effects on cougar movement across model structures, but the habitat cougar model did not explain their movements as well as the encounter model (ΔAIC = 4.70) that included avoidance of wolf kills. Cougars selected for rugged terrain (β_topographic roughness_ = 0.13, *P* < 0.001), forest cover (β_forest cover_ = 0.17, *P* < 0.001), and areas with less snow (β_snow depth_ = −0.18, *P* < 0.001; Table 1).

We did not detect an effect of wolf kill encounters on summer cougar movements (β_competitor kills_ = −0.15, *P* = 0.469), and this model accordingly had relatively less support (ΔAIC = 11.85) during this time. Otherwise, the determinants of seasonal cougar movements were surprisingly consistent.

### Interactions at Kill Sites.

There was extreme asymmetry in the location of wolf–cougar interactions as 41.8% of encounters (33 of 79) occurred at predicted cougar kills and only one encounter occurred at a wolf kill (based on our encounter metric distances). The interaction at the wolf kill did not appear to involve kleptoparasitism, as the wolf remained at the carcass while the cougar logged a single fix within 100 m before leaving the area. After filtering the cougar predation data to kills with known ungulate species that were available to the GPS-collared wolves, the proportion of elk, deer, and other ungulates species in cougar diets was 52.5%, 42.4%, and 5.1%, respectively (Fig. 5*D*). We documented wolves at 13.5% of the cougar-killed elk (21 of 156), 7.1% of the deer kills (9 of 126), and 6.7% of the other kills (1 of 15; Fig. 5*E*). Our binomial GLM estimated that the odds of wolves finding a cougar-killed elk were 2.0 times greater than a cougar-killed deer, although this relationship was only marginally statistically significant (*P* = 0.092; Fig. 5*E* and *SI Appendix*, Table S6 and *A*). Cougars were still present (within 250 m) at 38.1% (8 of 21) of the elk carcasses, compared to just 11.1% (1 of 9) of the deer carcasses (Fig. 5*E*). The odds of this probable kleptoparasitism occurring at a cougar-killed elk site were 6.8 times greater than a cougar-killed deer site, again with marginal significance (*P* = 0.07; *SI Appendix*, Table S6 and *A*). These patterns demonstrate that wolves actively kleptoparasitize rather than passively scavenge cougar kills.

## Discussion

Identifying the mechanisms that allow for the coexistence of apex carnivores with clear dominance hierarchies is critical to inform global carnivore restoration efforts. Using 9 y of contemporaneous GPS telemetry from wolves and cougars, we show that interactions among these reconstituted species are driven by asymmetric kleptoparasitism and intraguild killing, creating an enemies without benefits dynamic distinct from apex-mesocarnivore systems. Unlike mesocarnivores that balance scavenging opportunities with mortality risk at apex carnivore kills, subordinate apex carnivores like cougars suffer interference competition from dominant apex carnivores while handling their own kills. This fundamental difference emerges when subordinate carnivore hunting efficiency reduces their reliance on scavenging and dominant carnivore kleptoparasitism is enabled by body size and sociality. Interference competition at these conflict hotspots can be a powerful force in carnivore communities that excludes species from otherwise suitable habitat (e.g., wild dogs, cheetahs) (9, 10), but cougars in northern Yellowstone have persisted with no observed change in density despite the recolonization of wolves (15, 29). Understanding the mechanisms that have enabled their persistence requires expanding theory to explicitly incorporate variation in kleptoparasitism rates, intraguild killing, and dietary plasticity. This expansion is critical for carnivore restoration projects, where coexistence depends on reestablishing competitive hierarchies and resource partitioning after decades or centuries of absence.

The asymmetry of wolf and cougar interactions was striking. Wolves exhibited strong selection for cougar kills (2.1 times baseline) which intensified dramatically when cougars were present (7.5 times baseline; Fig. 4*C*). In contrast, cougars actively avoided wolf kills and never kleptoparasitized wolf kills (Fig. 4*B* and Table 1). Wolf dominance over cougars stems from their sociality, as wolves in Yellowstone average 10 individuals per pack (14). However, wolves maintained this advantage during summer despite reduced pack cohesiveness and competition over kleptoparasitic opportunities with black bears (*Ursus americanus*) and grizzly bears (*Ursus arctos horribilis*) (14, 15). Our smaller sample size of summer kleptoparasitic interactions limited seasonal comparisons, but wolf selection for such encounters was marginally stronger compared to winter (Fig. 4 *B* and *C*). Stronger selection for kleptoparasitism in summer could be driven by multiple mechanisms—smaller carcass sizes and bear kleptoparasitism reducing the temporal opportunity to obtain carrion, or behavioral changes of wolves driven by their lower biomass acquisition rates and higher cougar kill rates (16, 17). Across seasons, the long-distance movements of wolves—whose mean step length was 3.4 times greater than that of cougars (*SI Appendix*, Fig. S2)—likely allow them to find and usurp cougar kills opportunistically while engaged in other behaviors (e.g., hunting, territoriality, etc.). However, wolves were also attracted to cougars themselves even when controlling for kill sites (Fig. 4*A*), suggesting the possibility of wolves actively following cougars (e.g., using olfaction) for potential kleptoparasitic opportunities.

Subordinate carnivores must exhibit behavioral adaptations to persist under such asymmetric competitive costs. Our findings reveal that cougar coexistence with wolves depends on the complementary strategies of spatial refugia and dietary plasticity. Cougar use of topographic roughness increased with wolf proximity (Fig. 4*D*), and they strongly selected for this habitat and forest cover in general (Table 1). These movements are consistent with previous findings that cougars increased their selection for shorter distances to escape terrain (e.g., climbable trees or rocky outcroppings) in response to wolf reintroductions (15). This distinction, where cougars simply require key habitat features nearby to evade wolves, may explain why cougars did not increase their selection for forest cover when wolves were close (Table 1). If cougar kills occurred in open habitat, then reluctance to leave such areas when wolves usurped kills could register as selection for less forest cover, even if the presence of some trees was critical (e.g., Fig. 2*A*, *SI Appendix, A*, and Movie S2). Nonetheless, spatial refugia alone cannot eliminate the risks of kleptoparasitism. Dietary plasticity emerges as a critical secondary mechanism.

Cougar dietary shifts to smaller-bodied deer altered the competitive landscape between these apex carnivores. Shifting to smaller-bodied deer reduces handling times (16) and thus the temporal window for detection and usurpation by wolves. Field investigations confirmed prey body size was a key mediator of this relationship, as wolves kleptoparasitized elk kills six times more frequently than deer kills (Fig. 5*E* and *SI Appendix*, Table S6 and *A*). Cougar dietary changes were likely driven by the longitudinal decline of elk abundance in northern Yellowstone (Fig. 5*A*). Deer consumption was over four times greater in our study compared to 1998 to 2005 when elk abundance was higher (Fig. 5*C* and *SI Appendix*, Table S2 and *A*) and primarily consisted of forest-dependent mule deer (30) whose abundance likely remained relatively stable over the last 25 y (31). In contrast, wolves responded to the elk decline by increasing their scavenging and kill rates of bison (*Bison bison*; Fig. 5*B*) (32), a grassland-associated species whose abundance increased substantially over this period (33). These divergent dietary shifts reduced niche overlap by over 15% and likely reduced encounter rates because of alternative prey occupying different habitats. The effectiveness of spatial and dietary strategies is evident in cougar population stability over the last 25 y (Fig. 5*A*), demonstrating that subordinate apex carnivores can persist when landscape heterogeneity and prey diversity enable complementary mechanisms for coexistence.

Prey diversity spanning body size classes appears critical for apex carnivore coexistence through multiple pathways. Notably, social apex carnivore group sizes depend on large prey availability (34). Because group-living yields diminishing per-capita returns when prey are shared, social carnivores must compensate by hunting prey that are too large to kill individually or exhibit fission–fusion dynamics that maintain social benefits (e.g., territorial defense) while allowing for efficient foraging (14, 34, 35). The impacts sociality has on hunting tradeoffs then create opportunities for partitioning. As solitary ambush predators, cougars are substantially more efficient than wolves. They can successfully capture prey in over 80% of attempts (36), while wolves in Yellowstone succeed in less than 15% of hunts (37), contributing to cougar per-capita kill rates that are double those of wolves (15, 38). Thus, when densities are similar, cougars are superior exploitation competitors, particularly for smaller-bodied prey, while wolves are superior interference competitors (14). Prey diversity plays a key role in shaping these divergent strategies that reflect trade-offs between hunting efficiency and competitive dominance.

As wolves continue recolonizing western North America, our framework predicts that cougar persistence will depend on the availability of deer and escape terrain, with competitive exclusion most likely in low-diversity systems dominated by large ungulates (e.g., elk, feral horses) and open habitat. Exclusion risk stems from the energetic cost of kleptoparasitic encounters that also create conflict hotspots where intraguild killing can occur. Of the twelve known-fate mortalities of GPS-collared cougars during our study, two (17%) resulted from wolves finding cougars on elk kills in areas lacking escape terrain (Fig. 2*C*). This intraguild killing rate is similar to the four (20%) adult/subadult cougar mortalities that were attributed to wolves in northern Yellowstone from 1998 to 2005 (15). In contrast, cougars rarely kill wolves. None of the ninety monitored wolf mortalities during our study were attributed to cougars, though two dispersing wolves were killed by cougars outside of Yellowstone National Park in 2000 and 2003, and additional fatalities have occurred elsewhere when wolves are solitary or in pairs (15, 39). Yet, so long as wolves maintain pack structure, they will likely retain competitive dominance via kleptoparasitism and intraguild killing. These asymmetric interactions can generate complex ecosystem consequences. If kleptoparasitism increases cougar kill rates, their combined predation pressure could become superadditive; conversely, wolf-caused cougar mortality in systems with limited escape terrain could reduce cougars impact on prey. In northern Yellowstone, cougar distributions may be partially structured by intraguild killing risk forcing increased use of areas near escape terrain, yet cougar abundance appears resilient to such competition (15, 29). Cougar kill rates show reduced sensitivity to kleptoparasitism following the dietary shift toward deer (16), contrasting 1998 to 2005 when higher elk consumption elevated kill rates due to wolf and bear kleptoparasitism (15). This dietary plasticity by cougars can therefore reduce interference competition for cougars, as well as the effects they have on prey populations.

Our results demonstrate that the reintroduction of wolves to Yellowstone revived a competitive hierarchy with cougars that is structured by asymmetric kleptoparasitism and intraguild killing, producing an enemies without benefits dynamic. Group-living wolves were consistently dominant to solitary cougars across seasons, yet cougars persisted through a combination of spatial refugia and dietary plasticity. Landscape structure provided essential escape opportunities, while a shift toward smaller-bodied deer reduced handling times and consequently the likelihood of wolf encounters. Together, this suggests that coexistence among apex carnivores may depend less on overall prey abundance and more on prey diversity spanning body size classes that facilitates niche partitioning and mechanisms to reduce interference competition. More broadly, incorporating kleptoparasitism, intraguild killing, and dietary plasticity into frameworks of apex carnivore interactions provides a predictive basis for understanding how the restoration of multiple top predators will affect one another and, ultimately, the structure of ecological communities.

## Supplementary Material

Appendix 01 (PDF)

Movie S1. Video clip of wolves kleptoparasitizing a cougar kill. The first clip of the video shows the cougars who appear to detect the incoming wolf pack before they scatter. The second clip, 15 minutes later, shows the wolves feeding on the bull elk carcass.

Movie S2. Video of animated GPS data for a cougar (blue points/lines) and wolf (red points/lines) interaction at a cow elk kill made by the cougar. This interaction is the same as Fig. 2A of the main text.

## Data Availability

Data and code data have been deposited in Data and Code: Diets, dominance hierarchies, and kleptoparasitism drive asymmetrical interactions between wolves and cougars (10.5281/zenodo.15353246) (40).
