# Supplementary material for: Diets, dominance hierarchies, and kleptoparasitism drive asymmetrical interactions between wolves and cougars
Source: Proc Natl Acad Sci U S A. 2026 Jan 26;123(6):e2511397123. doi: 10.1073/pnas.2511397123 (PMC12890783; doi:10.1073/pnas.2511397123)
Supplement: Supplementary file 1 — Appendix 01 (PDF) [file pnas.2511397123.sapp.pdf]

PNAS

Supporting Information for

Diets, dominance hierarchies, and kleptoparasitism drive asymmetrical interactions between wolves and cougars.

Wesley Binder, Joel S. Ruprecht, Jack Rabe, Matthew C. Metz, Rebecca Hutchinson, Daniel R. Stahler, Taal Levi.

Wesley Binder
Email: wesley.binder@oregonstate.edu

This PDF file includes:

Appendix A
- Supporting text
- Figures S1 to S2
- Tables S1 to S6
- Legends for Movies S1 to S2
- SI References: Appendix A

Appendix B
- Wolf Random Forests
- Cougar Random Forests
- SI References: Appendix B

Appendix A

Supporting Information Text

Text S1. Data Collection. All capture and handling was conducted in accordance with the National Park Service protocols (IACUC permit IMR_YELL_Smith_wolves_2012; IMR_YELL_Stahler_Cougar_2018_A1). Data used for our analyses were restricted to 2016 – 2024 as the third phase of cougar collaring efforts in Yellowstone National Park began in December 2015. GPS data from juvenile cougars that were still dependent offspring were omitted, as well as wolves that did not spend at least 10% of the winter in northern Yellowstone (delineated by the Northern Range boundary [main text Fig. 1]). Wolves were assigned to packs through repeated observations by ground and aerial crews made during our predation monitoring periods, including daily attempts by each crew during the winter (1).

Text S2. Predation Monitoring. Winter predation monitoring was done for approximately 30 days per individual during early winter (mid-November through mid-December) and late winter (March), while summer predation monitoring was done for variable lengths of time within the months of May through July. Wolf clusters were identified as a minimum of two GPS fixes that occurred within 100m and three days of each other, while cougar clusters were identified as a minimum of two GPS fixes that occurred within 200m and six days of one another. Wolf GPS data used in the clustering algorithm was retained at one-hour fix rates in the winter and 30-minute fix rates in the summer, while all cougar GPS data was filtered to only one fix every three hours following Knopff et al. (2009) (2). This distinction reflects the differences in wolf and cougar handling times as pack-living wolves consume their prey quickly compared to solitary cougars, especially during summer (1). We used the "GPSeqClus" R package (3) to generate clusters and subsequently searched these locations for prey remains after the individuals left the area.

Winter predation monitoring for wolves also included ground observations and aerial flights (1). Ground observations were conducted by crews who were assigned to individual wolf packs, and these crews were tasked with observing wolves during daylight hours, if possible. This was primarily accomplished by using spotting scopes at ideal vantage points near roads. Aerial flights were conducted every day, weather permitting. Flights were conducted during the morning hours when wolves are most active, and attempts were made to observe each pack that was within the park's boundary at the time. Occasionally, cougar kills were observed during flights to count kittens associated with collared females and such kills were included in our predation dataset.

To calculate Pianka's overlap index (4) for dietary overlap between wolf and cougar, only ungulate species were used because smaller prey species were assumed to be an unlikely source of exploitation or interference competition between these apex carnivores. We combined deer species (mule [*Odocoileus hemionus*] and white-tailed deer [*Odocoileus virginianus*]) for all applicable analyses due to the high number of unknown deer species found, however, mule deer occur at much higher densities (5) and few white-tailed deer were confirmed to be predated upon.

Text S3. Integrated Step-Selection Functions. Wolf and cougar GPS collars were programmed to record one-hour fix rates during early winter (mid-November through mid-December), late winter (March), and spring-summer (May-July) when predation monitoring occurred. Wolf collars were programmed to collect 30-minute fix rates during summer predation periods, however, we filtered such GPS data to hourly fixes for our movement analyses in order to maintain consistent fix rates across seasons and carnivore species. Environmental covariates included an index of topographic roughness and percent tree cover (6), each at a 250 × 250 m resolution, as well as snow depth at a 1 km^2 resolution (7). We included these particular environmental covariates as previous work has demonstrated their strong influence on both wolf and cougar movements (1, 5, 6) and we chose resolutions to match the resolution of competitor encounters, when possible. All habitat covariates were scaled to have a mean of zero and a standard deviation of one.

When GPS-collared wolves spent at least 70% of a given winter month in the same pack as another GPS-collared wolf, one wolf was omitted from the movement analyses (for that period only) to avoid pseudoreplication occurring from non-independence. Wolves were either omitted randomly, or in the event one wolf left the pack, the individual that better represented pack

movements (i.e., the wolf that remained in the pack) was retained. This filtering was not done during the summer months as wolf packs are less cohesive then (1).

The GPS fixes of one cougar were omitted for a two-week period due to a predation event that occurred in the same location as a wolf kill three days prior. Field crews that investigated this site found two elk kills in close proximity. The GPS data was omitted because 1) cougar iSSF models that did not remove these data showed erroneous cougar selection of wolf kills because the cougar spent two weeks exclusively at this location and 2) such happenstance of multiple carnivore kills in the same location during the same month is presumed to be very rare. We examined other wolf-cougar interactions at known kill sites to determine if additional instances of multiple kills in close proximity occurred that might confound model results, but did not find any.

Text S4. Annual Density Estimates. Wolf densities were estimated by dividing the total number of wolves in Northern Range packs in December of each year by the area of the Northern Range (Fig. 1 [main text]). The total number of wolves were estimated via repeated ground and aerial observations (1). Cougar densities were estimated by Ruth (2019) using intensive capture and collaring efforts (1998-2003) and by Anton (2020) using non-invasive snow tracking surveys and spatial capture-recapture models (5, 8). Elk densities were estimated by aerial flights conducted each winter following protocols described in Tallian (2017) and are specific to the portion of the Northern Range that falls within Yellowstone National Park as their densities vary substantially inside and outside of the park (Fig. 1 [main text]) (9).

Text S5. Interactions at Kill Sites. To estimate which cougar kills of known ungulate species were available to the GPS collared wolves included in the iSSF analysis, minimum convex polygons were constructed using their GPS fixes for the applicable one-month predation monitoring period. All cougar kills that were made during these periods, as well as one week prior, and fell within a wolf's minimum convex polygon were considered "available". The additional analysis of "possible kleptoparasitism" that included a 1km buffer was used here to increase our sample sizes of potential wolf-cougar interactions occurring at kill sites with known prey species, as these were low when using the shorter buffer of 250m. Results from the binomial GLMs associated with these interactions are below (Table S6).

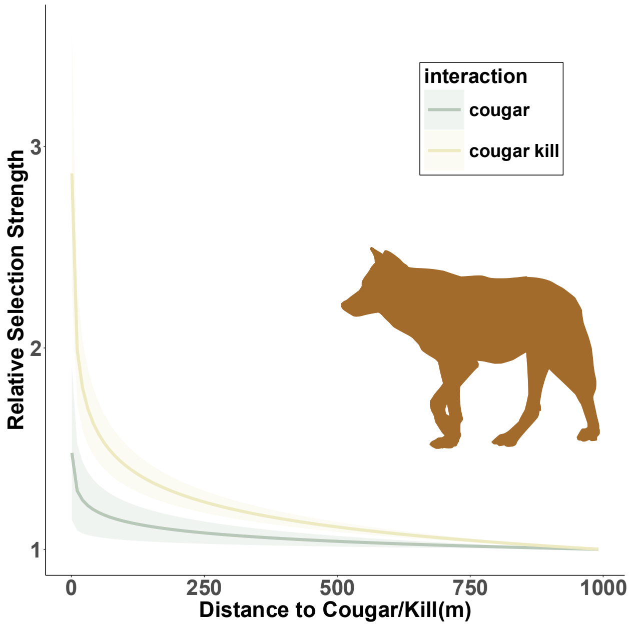

Fig. S1. (A) Wolf relative selection strength (RSS) for cougars and cougar kills as distance increases (based on the "attraction" metric estimates). The RSS measures the extent to which wolves are more likely to select for a cougar/cougar kill at variable distances (0-1000m) compared to a cougar/cougar kill at a reference distance of 1000m away.

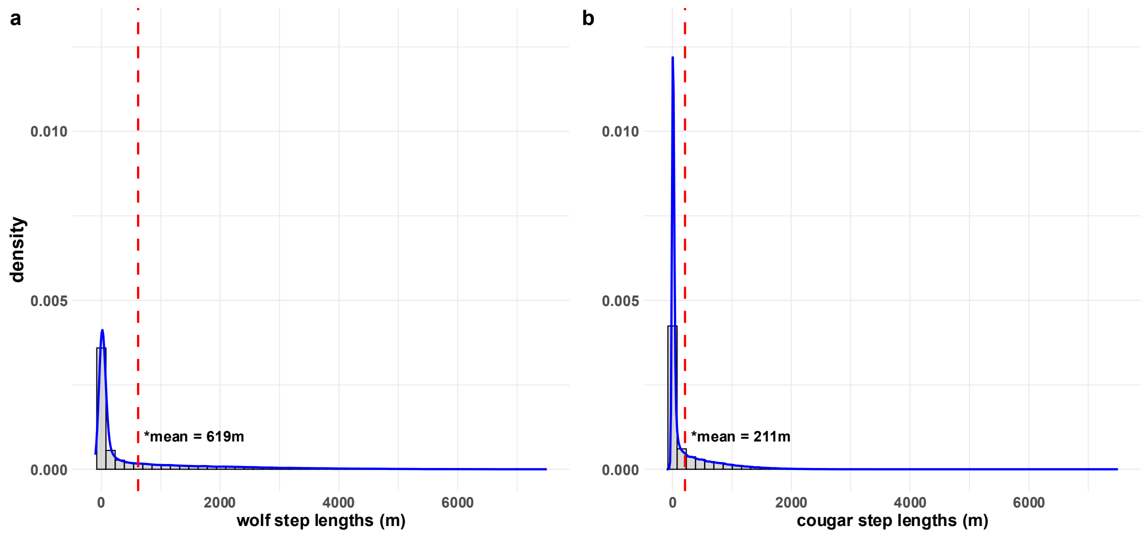

Fig. S2. (A) Wolf and (B) cougar steps length distributions, with dashed red lines depicting the mean step length of each species using 1-hour fix rates.

Table S1. Variation in the “encounter: competitor present” distance buffers (within which encounters are said to have occurred) to show how different distances affect inferences related to wolf kleptoparasitism of cougar kills. We opted for the 250m and 100m distance thresholds (bolded) for competitors and competitor kills (here cougar and cougar kills), respectively, to ensure encounters were very likely to have occurred. The cougar kills used here are the predicted kills from the best winter and summer random forest models.

Carnivore	“Competitor Present” Distances (m): Cougar / Cougar Kill	Total # of Encounters	β	SE	P
wolf	200 / 100	24	0.81	0.35	0.022
	**250 / 100**	**33**	**1.26**	**0.32**	**<0.001**
	300 / 100	35	1.30	0.30	<0.001
	250 / 50	19	1.53	0.39	<0.001
	250 / 150	42	0.99	0.29	<0.001
	250 / 200	46	0.73	0.28	0.010
	300 / 150	44	0.92	0.28	<0.001
	300 / 200	48	0.96	0.27	<0.001

Table S2. Binomial GLMs parameter estimates for wolf and cougar prey use changes by research phase (1998-2005 and 2016-2024).

Carnivore	Prey Species	Covariate	β	SE	P
wolf	deer	intercept (1998-2005)	-4.32	0.32	< 0.001
		2016-2024	2.19	0.34	< 0.001
	elk	intercept (1998-2005)	3.01	0.17	< 0.001
		2016-2024	-2.45	0.19	< 0.001
	bison	intercept (1998-2005)	-3.43	0.21	< 0.001
		2016-2024	2.26	0.22	< 0.001
cougar	deer	intercept (1998-2005)	-1.75	0.14	< 0.001
		2016-2024	1.44	0.16	< 0.001
	elk	intercept (1998-2005)	1.38	0.12	< 0.001
		2016-2024	-1.28	0.15	< 0.001

Table S3. Wolf and cougar parameter estimates from each iSSF model (arranged by species and ΔAIC). The combined winter and summer data were used here.

Carnivore	Model	Covariate	β	SE	P
wolf	habitat + "attraction" ΔAIC = 0	competitors	-0.06	0.02	0.003
		competitor kill	-0.15	0.02	< 0.001
		topographic roughness	-0.12	0.01	< 0.001
		forest cover	-0.06	0.01	< 0.001
		snow depth	-0.24	0.02	< 0.001
	habitat + "encounters: competitor present" ΔAIC = 38.91	competitors	0.11	0.17	0.534
		competitor kill	0.75	0.11	< 0.001
		competitor present	1.26	0.33	< 0.001
		topographic roughness	-0.12	0.01	< 0.001
		forest cover	-0.06	0.01	< 0.001
		snow depth	-0.26	0.02	< 0.001
	habitat + "encounters" ΔAIC = 51.40	competitors	0.37	0.15	0.011
		competitor kill	0.88	0.10	< 0.001
		topographic roughness	-0.12	0.01	< 0.001
		forest cover	-0.06	0.01	< 0.001
		snow depth	-0.26	0.02	< 0.001
	habitat only ΔAIC = 126.36	topographic roughness	-0.12	0.01	< 0.001
		forest cover	-0.06	0.01	< 0.001
		snow depth	-0.26	0.02	< 0.001
cougar	habitat + "encounters" ΔAIC = 0	competitors	0.17	0.17	0.311
		competitor kill	-0.47	0.17	0.008
		topographic roughness	0.13	0.01	< 0.001
		forest cover	0.17	0.01	< 0.001
		snow depth	-0.18	0.03	< 0.001
	habitat + "attraction: habitat interaction" ΔAIC = 1.45	competitors	0.03	0.07	0.639
		competitors × topo	-0.01	0.01	0.036
		competitors × cover	0.02	0.01	0.026
		topographic roughness	0.24	0.05	< 0.001
		forest cover	0.04	0.06	0.563
		snow depth	-0.18	0.03	< 0.001
	habitat only ΔAIC = 4.70	topographic roughness	0.13	0.01	< 0.001
		forest cover	0.17	0.01	< 0.001
		snow depth	-0.18	0.03	< 0.001
	habitat + "attraction" ΔAIC = 8.24	competitors	0.02	0.05	0.717
		competitor kill	0.01	0.03	0.619
		topographic roughness	0.13	0.01	< 0.001
		forest cover	0.17	0.01	< 0.001
		snow depth	-0.18	0.03	< 0.001

Table S4. Wolf and cougar parameter estimates from each iSSF model (arranged by species and ΔAIC) with winter movements only.

Carnivore	Model	Covariate	β	SE	P
wolf	habitat + "attraction" ΔAIC = 0	competitors	-0.04	0.03	0.244
		competitor kill	-0.18	0.02	< 0.001
		topographic roughness	-0.07	0.01	< 0.001
		forest cover	-0.14	0.01	< 0.001
		snow depth	-0.21	0.02	< 0.001
	habitat + "encounters: competitor present" ΔAIC = 15.69	competitors	0.19	0.21	0.357
		competitor kill	0.82	0.13	< 0.001
		competitor present	0.93	0.38	0.014
		topographic roughness	-0.07	0.01	< 0.001
		forest cover	-0.14	0.01	< 0.001
		snow depth	-0.23	0.02	< 0.001
	habitat + "encounters" ΔAIC = 19.67	competitors	0.41	0.18	0.022
		competitor kill	0.92	0.12	< 0.001
		topographic roughness	-0.07	0.01	< 0.001
		forest cover	-0.14	0.01	< 0.001
		snow depth	-0.23	0.02	< 0.001
	habitat only ΔAIC = 79.03	topographic roughness	-0.07	0.01	< 0.001
		forest cover	-0.14	0.01	< 0.001
		snow depth	-0.23	0.02	< 0.001
cougar	habitat + "encounters" ΔAIC = 0	competitors	0.11	0.20	0.585
		competitor kill	-0.99	0.32	0.002
		topographic roughness	0.10	0.01	< 0.001
		forest cover	0.14	0.01	< 0.001
		snow depth	-0.18	0.03	< 0.001
	habitat + "attraction: habitat interaction" ΔAIC = 8.44	competitors	0.03	0.07	0.639
		competitors × topo	-0.02	0.01	0.021
		competitors × cover	0.01	0.01	0.355
		topographic roughness	0.29	0.08	< 0.001
		forest cover	0.06	0.10	0.567
		snow depth	-0.18	0.03	< 0.001
	habitat only ΔAIC = 8.57	topographic roughness	0.10	0.01	< 0.001
		forest cover	0.14	0.01	< 0.001
		snow depth	-0.18	0.03	< 0.001
	habitat + "attraction" ΔAIC = 11.56	competitors	0.02	0.07	0.802
		competitor kill	0.04	0.04	0.358
		topographic roughness	0.10	0.01	< 0.001
		forest cover	0.14	0.01	< 0.001
		snow depth	-0.18	0.03	< 0.001

Table S5. Wolf and cougar parameter estimates from each iSSF model (arranged by species and ΔAIC) with summer movements only. Snow depth was not included as a summer covariate.

Carnivore	Model	Covariate	β	SE	P
wolf	habitat + "attraction" ΔAIC = 0	competitors	-0.07	0.02	0.004
		competitor kill	-0.14	0.02	< 0.001
		topographic roughness	-0.18	0.01	< 0.001
		forest cover	-0.04	0.01	< 0.001
	habitat + "encounters: competitor present" ΔAIC = 31.88	competitors	-0.05	0.31	0.860
		competitor kill	0.57	0.21	0.006
		competitor present	2.31	0.67	< 0.001
		topographic roughness	-0.17	0.01	< 0.001
		forest cover	-0.04	0.01	< 0.001
	habitat + "encounters" ΔAIC = 41.56	competitors	0.32	0.25	0.206
		competitor kill	0.77	0.19	< 0.001
		topographic roughness	-0.17	0.01	< 0.001
		forest cover	-0.04	0.01	< 0.001
	habitat only ΔAIC = 54.49	topographic roughness	-0.17	0.01	< 0.001
		forest cover	-0.04	0.01	< 0.001
cougar	habitat + "attraction: habitat interaction" ΔAIC = 0	competitors	0.00	0.08	0.972
		competitors × topo	-0.03	0.01	< 0.001
		competitors × cover	0.00	0.01	0.674
		topographic roughness	0.43	0.07	< 0.001
		forest cover	0.16	0.09	0.066
	habitat only ΔAIC = 9.29	topographic roughness	0.16	0.01	< 0.001
		forest cover	0.20	0.01	< 0.001
	habitat + "encounters" ΔAIC = 11.85	competitors	0.30	0.30	0.323
		competitor kill	-0.15	0.21	0.469
		topographic roughness	0.16	0.01	< 0.001
		forest cover	0.20	0.01	< 0.001
	habitat + "attraction" ΔAIC = 13.22	competitors	0.01	0.08	0.884
		competitor kill	-0.01	0.04	0.801
		topographic roughness	0.16	0.01	< 0.001
		forest cover	0.20	0.01	< 0.001

Table S6. Results from the binomial GLMs used to estimate the odds of a GPS-collared wolf discovering cougar kills of known prey species (interaction type = cougar kill; wolf GPS fix <100m of kill), and interactions with such kills when the cougar was also within 250m of the wolf (interaction type = probable kleptoparasitism). We reported these results in the main text as both odds (exponentiated beta coefficients) and as probabilities (via the inverse logit transformation). We further include interactions with such kills when the cougar was also within 1km of the wolf here to document potential kleptoparasitism (interaction type = possible kleptoparasitism).

Carnivore	Interaction Type	Sample Sizes	Covariate	β	SE	P
wolf	cougar kill	9 of 126	intercept (deer)	-2.57	0.35	< 0.001
		21 of 156	elk	0.70	0.42	0.092
	possible kleptoparasitism	2 of 126	intercept (deer)	-4.13	0.71	< 0.001
		14 of 156	elk	1.81	0.77	0.018
	probable kleptoparasitism	1 of 126	intercept (deer)	-4.83	1.00	< 0.001
		8 of 156	elk	1.91	1.07	0.074

Movie S1 (separate file). Video clip of wolves kleptoparasitizing a cougar kill. The first clip of the video shows the cougars who appear to detect the incoming wolf pack before they scatter. The second clip, 15 minutes later, shows the wolves feeding on the bull elk carcass.

Movie S2 (separate file). Video of animated GPS data for a cougar (blue points/lines) and wolf (red points/lines) interaction at a cow elk kill made by the cougar. This interaction is the same as Fig. 2A of the main text.

SI References: Appendix A

1. D. W. Smith, D. R. Stahler, D. R. MacNulty, *Yellowstone Wolves: Science and Discovery in the World's First National Park* (University of Chicago Press, 2020).
2. K. Knopff H., A. Knopff A., M. Warren B., M. S. Boyce, Evaluating Global Positioning System Telemetry Techniques for Estimating Cougar Predation Parameters. *J. Wildl. Manag.* **73**, 586–597 (2009).
3. J. G. Clapp, J. D. Holbrook, D. J. Thompson, GPSeqClus: An R package for sequential clustering of animal location data for model building, model application and field site investigations. Methods Ecol. Evol. **12**, 787–793 (2021).
4. E. R. Pianka, The Structure of Lizard Communities. *Annu. Rev. Ecol. Syst.* **4**, 53–74 (1973).
5. T. Ruth, P. Buotte, M. Hornocker, *Yellowstone Cougars: Ecology Before And During Wolf Restoration* (University Press of Colorado, 2019).
6. M. Kohl T., *et al.*, Do prey select for vacant hunting domains to minimize a multi-predator threat? *Ecol. Lett.* **22**, 1724–1733 (2019).
7. National Operational Hydrologic Remote Sensing Center, Snow Data Assimulation System (SNODAS) Data Products at NSIDC. https://doi.org/10.7265/N5TB14TC. Deposited 2004.
8. C. Anton, "The Demography and Comparative Ethology of Top Predators in a Multi-Carnivore System," UC Santa Cruz. (2020).
9. A. Tallian, *et al.*, Predator foraging response to a resurgent dangerous prey. *Funct. Ecol.* **31**, 1418–1429 (2017).

Appendix B: Random Forests Predictive Kill Models

Background

Random forests (hereafter "RFs") are machine-learning classifiers that have been widely adopted by ecologists due to their predictive accuracy and ability to capture interactions between covariates (1). RFs leverage multiple classification trees, each one trained on bootstrap samples (random subsets of the data) that make predictions on the out-of-bag, or withheld, data. Each classification tree splits the data into increasingly homogeneous regions based on the response variable, using covariates to determine each split. RFs use only a randomly selected subset of covariates to make each split which are determined by the mtry parameter. By combining predictions across classification trees, RFs can improve predictive accuracy while minimizing overfitting.

Here, we used our field investigations of aggregations of wolf and cougar GPS fixes (hereafter "clusters") in conjunction with RFs to predict the presence or absence of a kill site at locations that were not searched by field crews. Information regarding cluster searches can be found in the methods of the main text under "Predation Monitoring". Only ungulate carcasses found in the field were classified as "kills" here, as smaller prey were omitted to reduce false positive predictions. For the wolf cluster dataset, only one wolf from a given pack was included to avoid pseudo-replicated movements that could bias predictions. When two or more wolves belonged to the same pack, one individual was either randomly selected to be retained or the wolf that best represented the pack was retained (i.e., the wolf that remained with the pack for the largest portion of the predation monitoring period). We removed wolf and cougar den site clusters from our datasets to avoid such non-kill clusters with long durations influencing predictions. When we documented competitor tracks and sign (e.g., cache site) at clusters with prey remains, we did our best to determine if the focal carnivore made the kill or scavenged it based on tracks and sign at the cluster site (e.g., track age, cache sites, carcass articulation, etc.). If the prey remains were thought to be scavenging of the competitor's kill, we removed such clusters from our datasets here.

Methods

Wolf and cougar cluster datasets were each randomly split into training (60%), validation (20%), and testing (20%) datasets. RFs were generated using the training dataset, each model incorporating different combinations of mtry values (two, three, and six) and cluster network distances (see "clus_network" below). We incorporated information on cluster networks because both wolves and cougars often travel long distances before making a kill, after which their movements often remain localized in the general area. Accordingly, we wanted to provide models with information regarding the timing of a cluster's initiation relative to the other clusters in the area.

The number of trees used for each RFs were kept at 500 since the out-of-bag error rates remained stable with an increasing number of trees (see OOB plots below). We included network distances as a covariate due to the foraging behaviors of each carnivore, where successful kills are often preceded by larger movements (i.e., foraging) and followed by shorter movements. We therefore wanted to leverage information regarding the start times of clusters relative to nearby clusters.

The best performing RFs were chosen based on the highest area under the receiver operating curve (AUC) values associated with the validation datasets. AUC is an index of predictive performance that balances true positive rates (sensitivity) with true negative rates (specificity) such that high AUC values correspond to both high sensitivity and specificity. After the best RFs were selected, their predictive accuracy was confirmed by plotting the AUC values associated with the testing datasets.

Two thresholds were compared for assigning kills based on the cluster-specific probabilities estimated by the RFs. One threshold was based on the maximum value of sensitivity plus specificity ("maxSenSpec"), while the other was based on a specificity of 0.95. We used the maxSenSpec threshold so long as the corresponding specificity was not below 0.95 in an attempt to minimize the number of false positives (i.e.,

incorrectly predicted kills) and thus conservatively estimate the effect of a competitor's kills on the focal individual's movements.

Predicted kill sites used in our movement analyses were derived by generating clusters, applying the RFs such that each cluster was assigned a kill probability, and then using the selected threshold to determine the presence of a kill. Clusters were generated for the winter predation monitoring periods (early winter [mid-November through mid-December] and late winter [March]) used in the movement analyses, as well as the 30 days preceding these periods for cougar clusters such that the resulting probable kills would be available for wolves to interact with immediately (as each kill was "available" for a competitor to interact with for 30 days). In contrast, we could not predict wolf kills outside of the predation monitoring periods due to insufficient fix rates.

The predicted kills were then compared to the competitor's predicted kills to determine if assigned kills were actually made by the competitor (e.g., if a wolf stole a cougar kill, the RFs may incorrectly predict a wolf kill). When any predicted kill occurred within 150m and 30 days after a predicted kill from a competitor, that kill was omitted.

Covariates

n_clus_locs: total number of GPS fixes at the cluster.

night_pts: total number of GPS fixes at the cluster that occur after sunset and before sunrise.

night_prop: proportion of GPS fixes at the cluster that are night_pts.

fidelity: proportion of GPS fixes at the cluster during the cluster duration (i.e., from the first fix to the last fix at the cluster).

avg_clus_dist: average distance between GPS fixes at the cluster.

visits: the number of times the individual came to the cluster (e.g., if and individual initiated a cluster, left and then came back one time, visits = 2)

d_last_clus: distance to the last cluster.

lighting: categorical time of day of the first GPS fix at the cluster (categories: dawn, day, dusk, and night).

d_creek: distance to the nearest creek or river.

curvature: curvature of the landscape at a 100m resolution (estimated by taking the mean of 10m resolution values).

topo250: index of topographic roughness at a 250m resolution.

cover250: index of forest cover at a 250m resolution.

season: early (mid-November through mid-December) or late winter (March).

clus_network: categorical indicator of cluster's start time relative to other cluster start times within the same network based on various distances (categories: shared-always first, shared-sometimes first, shared-never first, and never shared). Network distances used were 200m, 400m, 600m, 800m, and 1km.

pack.size (wolf only): the number of wolves in the pack of the focal individual.

demo_group (cougar only): categorical sex/reproductive status designation (subadult male [1-3 years old], male [>3 years old], subadult female [1-3 years old], solitary female [>3 years old], female with kittens <6 months old, and female with kittens >6 months old).

Wolf Random Forests

Winter

Dataset

A total number of 1662 wolf clusters were used for this analysis. This resulted in 997 clusters in the training dataset, 332 clusters in the validation dataset, and 333 clusters in the testing dataset.

Table S7. AUC values from the winter wolf RFs that vary by mtry and network distance values. The cluster data used here were created with 100m, 3-day space-time parameters. The model with the highest AUC value was used to predict kill sites and is provided in bold text here.

Network	MTRY	AUC Value
200m	2	0.810
200m	3	0.814
200m	6	0.814
400m	2	0.823
**400m**	**3**	**0.831**
400m	6	0.831
600m	2	0.830
600m	3	0.828
600m	6	0.828
800m	2	0.825
800m	3	0.820
800m	6	0.820
1km	2	0.830
1km	3	0.828
1km	6	0.828

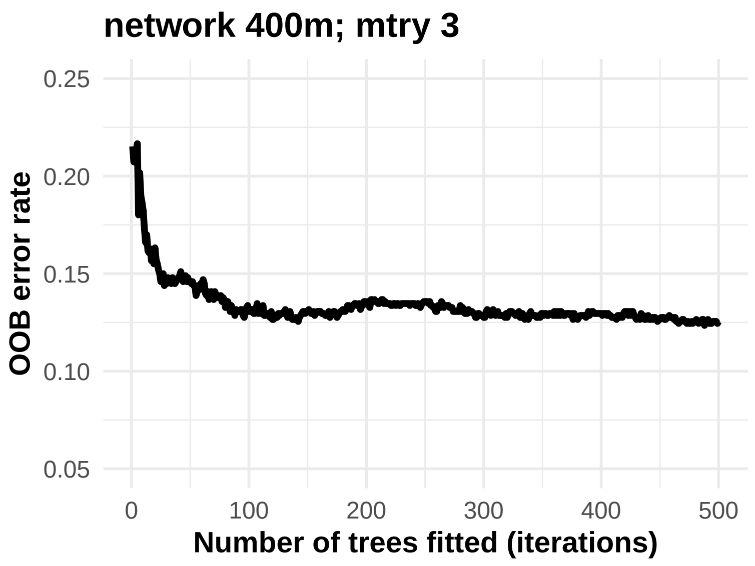

Figure S3. Out-of-bag error rates from the winter wolf RF model with the highest AUC value are plotted to look for potential overfitting as the number of trees increases.

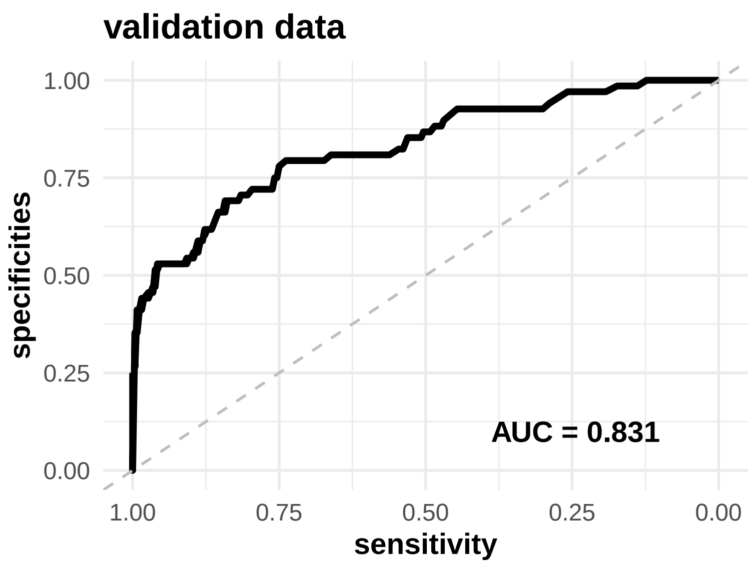

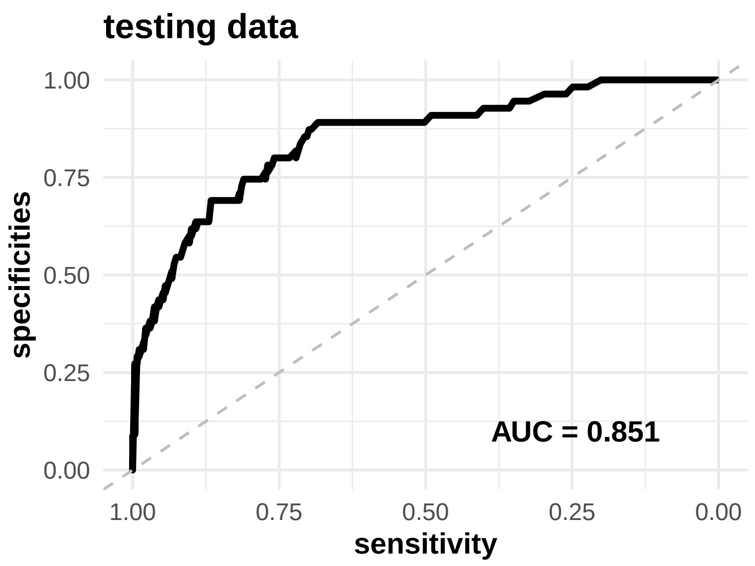

Figure S4. AUC plots from the best winter wolf model above. The top plot uses the validation data and the bottom plot uses the testing data, each of which are 20% of the cluster dataset that was not used to create the random forest model. Sensitivities (true positive rates) are plotted against specificities (true negative rates) as the threshold for assigning predictions varies.

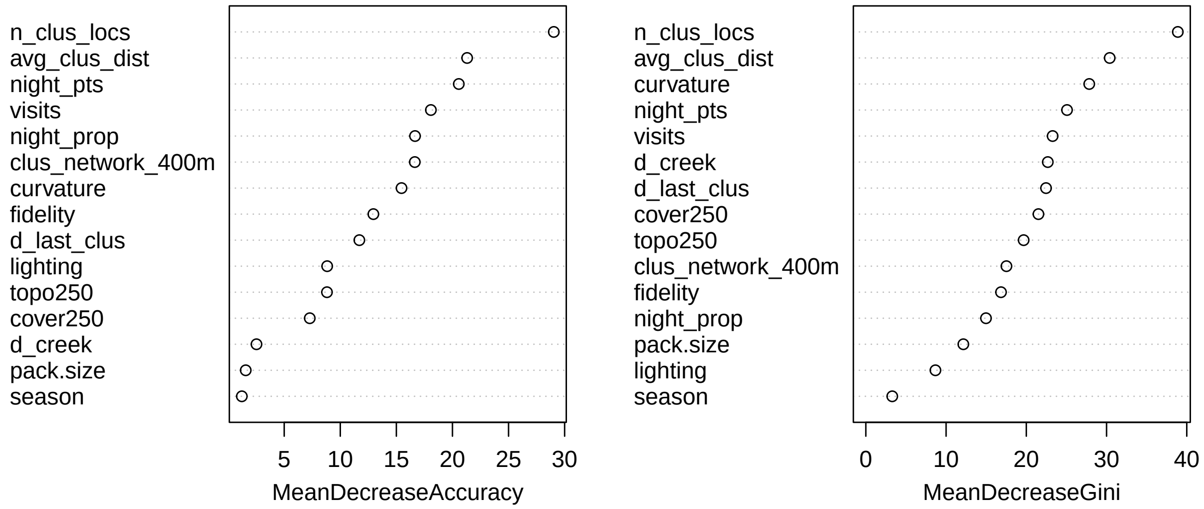

Figure S5. Variable importance plots using two metrics to assess the importance of each covariate in making predictions for the presence of kills at winter wolf clusters. First, the mean decrease in accuracy, which shows how the removal of a covariate affects the model's accuracy. Second, the mean decrease in Gini, which demonstrates how much each covariate contributes to reducing node impurity (Gini index), where higher values represent greater influence in splitting the data.

Thresholds for Classification

Here, we compare two thresholds to use for determining which clusters will be classified as kills based on the probabilities generated by the RFs. The first threshold ("maxSenSpec") is set based on the maximum value of sensitivity (true positive rate) plus specificity (true negative rate). The second threshold is set based on a specificity of 0.95 which is the lowest specificity we wanted to use in order to reduce false-positive predictions. We therefore decided to use the threshold based on the maximum sensitivity and specificity value unless the specificity for that threshold was less than 0.95.

```
## maxSenSpec sensitivity: 0.891

## maxSenSpec specificity: 0.684

## maxSenSpec threshold: 0.127

## specificity of 0.95 threshold: 0.418
```

Table S8. Confusion matrix of the winter wolf RF predictions for the testing dataset using the applicable threshold described above (here, the specificity of 0.95 threshold).

	Count
kill found; correct prediction	25
kill found; wrong prediction	30
no kill; correct prediction	255
no kill; wrong prediction	14

Final Model

The final model we use leverages all the cluster data along with the parameter values (mtry and network distance) used in the model above with the highest AUC value.

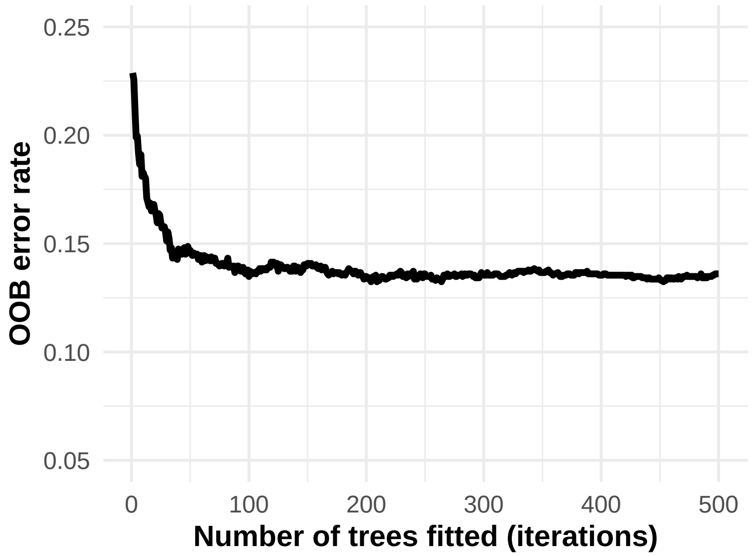

Figure S6. OOB error rates are plotted to check for potential overfitting of the final winter wolf RF model.

Wolf Random Forests

Summer

Dataset

A total number of 1002 wolf clusters were used for this analysis. This resulted in 601 clusters in the training dataset, 200 clusters in the validation dataset, and 201 clusters in the testing dataset.

Table S9. AUC values from the summer wolf RFs that vary by mtry and network distance values. The cluster data used here were created with 100m, 3-day space-time parameters. The model with the highest AUC value was used to predict kill sites and is provided in bold text here.

Network	MTRY	AUC Value
200m	2	0.801
200m	3	0.808
200m	6	0.808
400m	2	0.791
400m	3	0.793
400m	6	0.793
600m	2	0.799
600m	3	0.803
600m	6	0.803
**800m**	**2**	**0.810**
800m	3	0.799
800m	6	0.799
1km	2	0.805
1km	3	0.796
1km	6	0.796

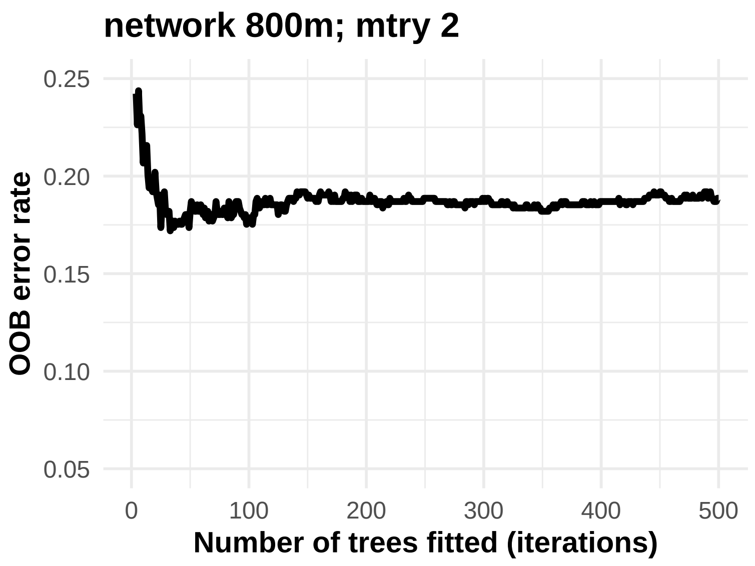

Figure S7. Out-of-bag error rates from the summer wolf RF model with the highest AUC value are plotted to look for potential overfitting as the number of trees increases.

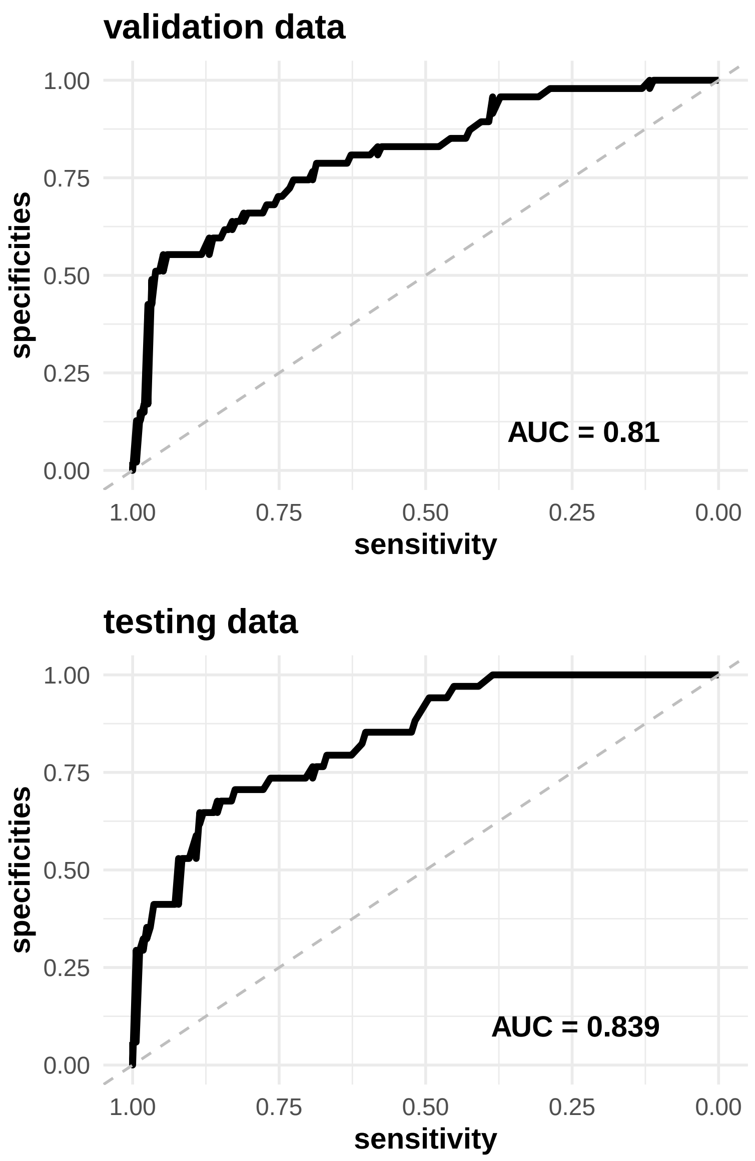

Figure S8. AUC plots from the best summer wolf model above. The top plot uses the validation data and the bottom plot uses the testing data, each of which are 20% of the cluster dataset that was not used to create the random forest model. Sensitivities (true positive rates) are plotted against specificities (true negative rates) as the threshold for assigning predictions varies.

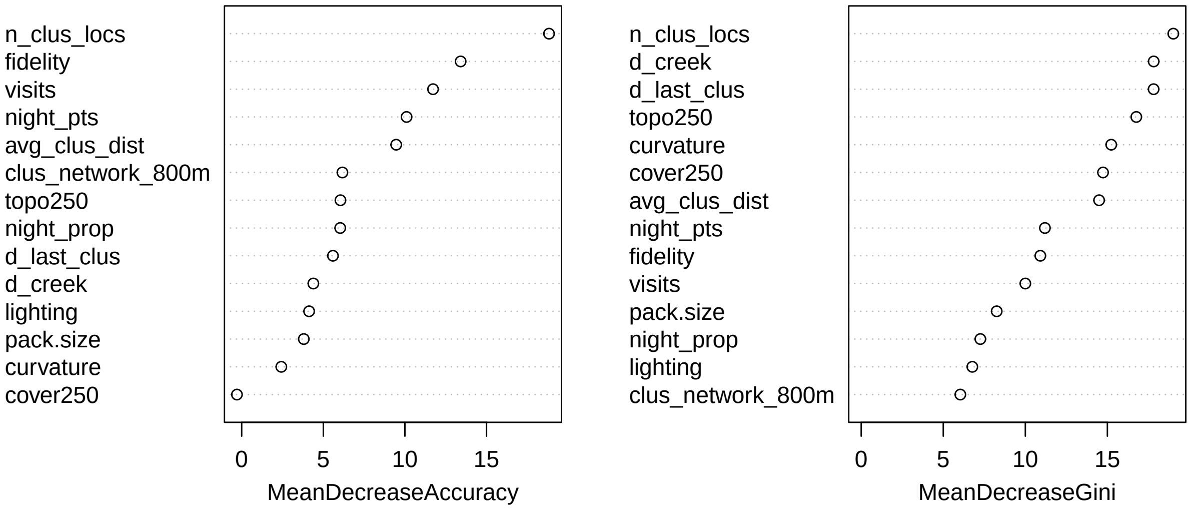

Figure S9. Variable importance plots using two metrics to assess the importance of each covariate in making predictions for the presence of kills at summer wolf clusters. First, the mean decrease in accuracy, which shows how the removal of a covariate affects the model's accuracy. Second, the mean decrease in Gini, which demonstrates how much each covariate contributes to reducing node impurity (Gini index), where higher values represent greater influence in splitting the data.

Thresholds for Classification

Here, we compare two thresholds to use for determining which clusters will be classified as kills based on the probabilities generated by the RFs. The first threshold ("maxSenSpec") is set based on the maximum value of sensitivity (true positive rate) plus specificity (true negative rate). The second threshold is set based on a specificity of 0.95 which is the lowest specificity we wanted to use in order to reduce false-positive predictions. We therefore decided to use the threshold based on the maximum sensitivity and specificity value unless the specificity for that threshold was less than 0.95.

```
## maxSenSpec sensitivity: 0.647
```

```
## maxSenSpec specificity: 0.886
```

```
## maxSenSpec threshold: 0.221
```

```
## specificity of 0.95 threshold: 0.261
```

Table S10. Confusion matrix of the summer wolf RF predictions for the testing dataset using the applicable threshold described above (here, the specificity of 0.95 threshold).

	Count
kill found; correct prediction	14
kill found; wrong prediction	20
no kill; correct prediction	157
no kill; wrong prediction	9

Final Model

The final model we use leverages all the cluster data along with the parameter values (mtry and network distance) used in the model above with the highest AUC value.

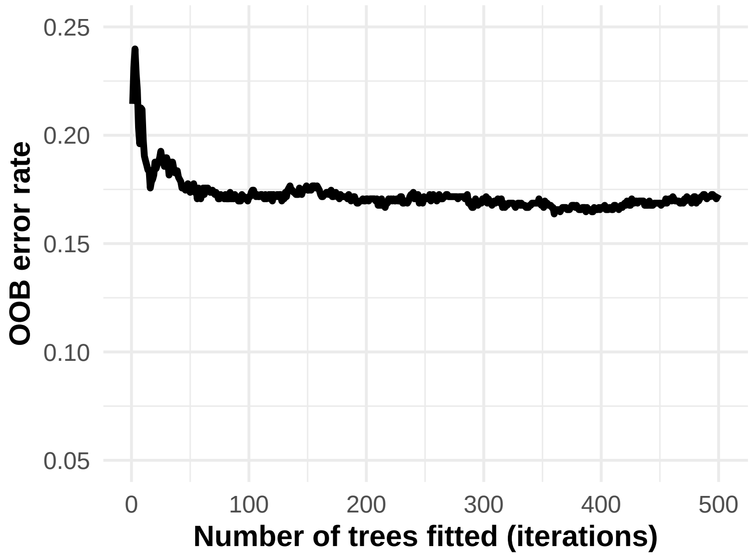

Figure S10. OOB error rates are plotted to check for potential overfitting of the final summer wolf RF model.

Cougar Random Forests

Winter

Here, we regenerated cougar clusters with 100m, three-day parameters and compared them to the original 200m, six-day parameters to determine if this would improve predictive performance.

Dataset

A total number of 586 cougar clusters were used for this analysis. This resulted in 352 clusters in the training dataset, 117 clusters in the validation dataset, and 117 clusters in the testing dataset.

Table S11. AUC values from the winter cougar RFs that vary by mtry and network distance values. The cluster data used here were created with 200m, 6-day cluster parameters (which were used to generate clusters searched by field crews and reduce the likelihood of kills being missed for kill rate estimates) and 100m, 3-day parameters (reduced space-time parameters thought to increase predictive accuracy). The model with the highest AUC value was used to predict kill sites and is provided in bold text here.

100m, 3-day cluster parameters

Network	MTRY	AUC Value
**200m**	**2**	**0.982**
200m	3	0.971
200m	6	0.971
400m	2	0.976
400m	3	0.974
400m	6	0.974
600m	2	0.975
600m	3	0.972
600m	6	0.972
800m	2	0.972
800m	3	0.970
800m	6	0.970
1km	2	0.974
1km	3	0.975
1km	6	0.975

200m, 6-day cluster parameters

Network	MTRY	AUC Value
200m	2	0.920
200m	3	0.912
200m	6	0.912
400m	2	0.914
400m	3	0.914
400m	6	0.914
600m	2	0.914
600m	3	0.909
600m	6	0.909
800m	2	0.909
800m	3	0.908
800m	6	0.908
1km	2	0.920
1km	3	0.914
1km	6	0.914

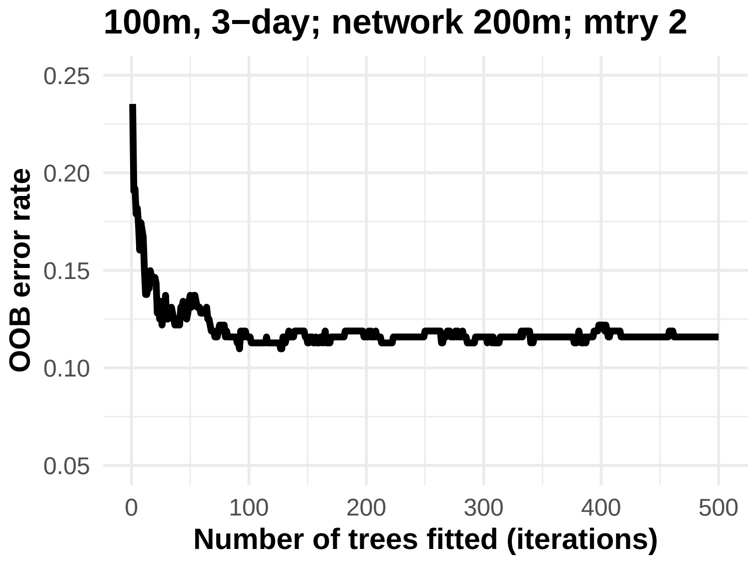

Figure S11. Out-of-bag error rates from the winter cougar RF model with the highest AUC value are plotted to look for potential overfitting as the number of trees increases.

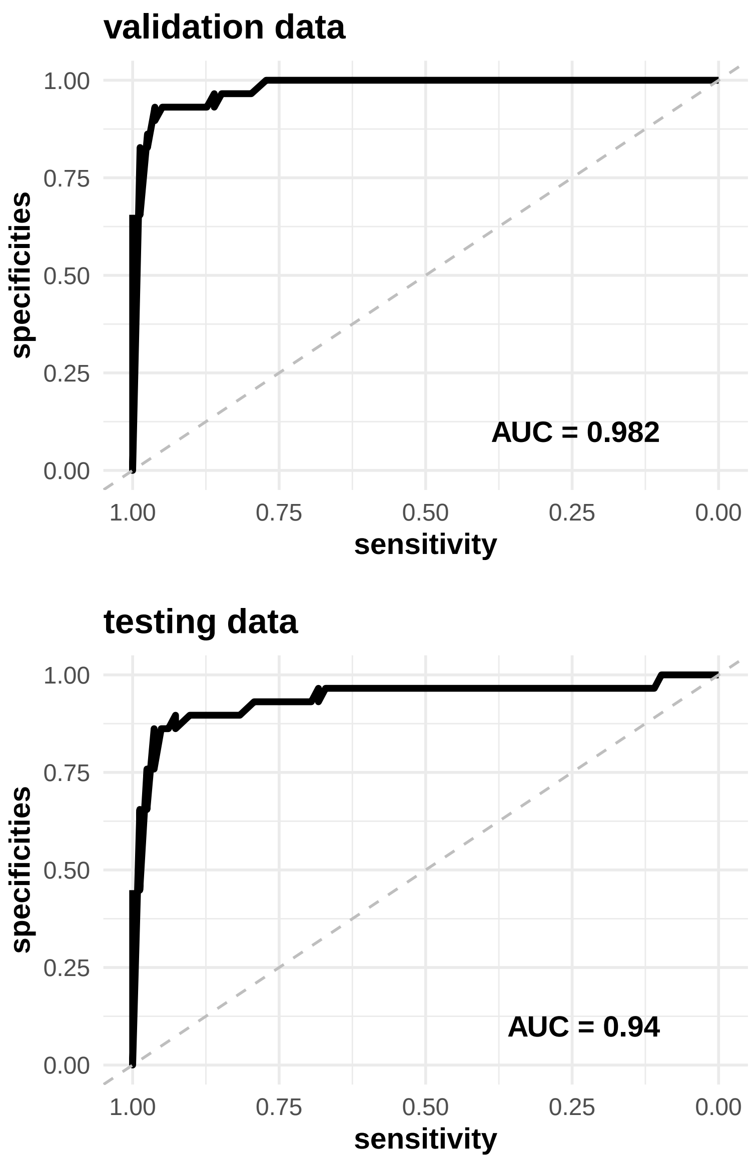

Figure S12. AUC plots from the best winter cougar model above. The top plot uses the validation data and the bottom plot uses the testing data, each of which are 20% of the cluster dataset that was not used to create the random forest model. Sensitivities (true positive rates) are plotted against specificities (true negative rates) as the threshold for assigning predictions varies.

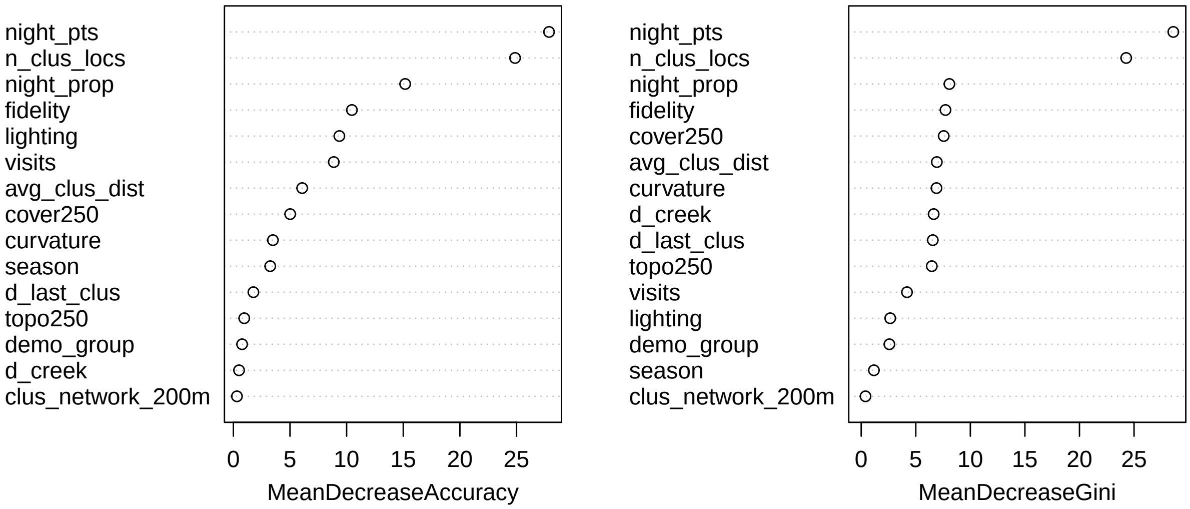

Figure S13. Variable importance plots using two metrics to assess the importance of each covariate in making predictions for the presence of kills at winter cougar clusters. First, the mean decrease in accuracy, which shows how the removal of a covariate affects the model's accuracy. Second, the mean decrease in Gini, which demonstrates how much each covariate contributes to reducing node impurity (Gini index), where higher values represent greater influence in splitting the data.

Thresholds for Classification

Here, we compare two thresholds to use for determining which clusters will be classified as kills based on the probabilities generated by the RFs. The first threshold ("maxSenSpec") is set based on the maximum value of sensitivity (true positive rate) plus specificity (true negative rate). The second threshold is set based on a specificity of 0.95 which is the lowest specificity we wanted to use in order to reduce false-positive predictions. We therefore decided to use the threshold based on the maximum sensitivity and specificity value unless the specificity for that threshold was less than 0.95.

```
## maxSenSpec sensitivity: 0.862

## maxSenSpec specificity: 0.963

## maxSenSpec threshold: 0.316

## specificity of 0.95 threshold: 0.291
```

Table S12. Confusion matrix of the winter cougar RF predictions for the testing dataset using the applicable threshold described above (here, the maxSenSpec threshold).

	Count
kill found; correct prediction	25
kill found; wrong prediction	4
no kill; correct prediction	79
no kill; wrong prediction	3

Final Model

The final model we use leverages all the cluster data along with the parameter values (cluster space and time parameters, as well as mtry and network distance) used in the model above with the highest AUC value.

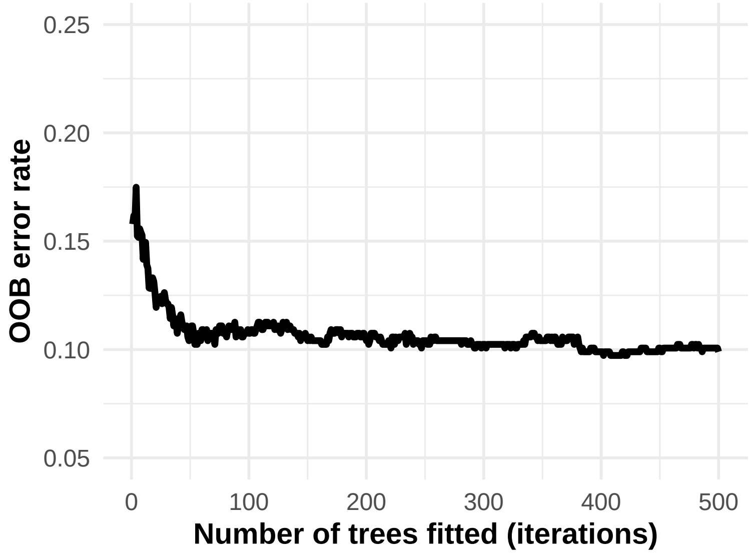

Figure S14. OOB error rates are plotted to check for potential overfitting of the final winter cougar RF model.

Cougar Random Forests

Summer

Here, we regenerated cougar clusters with 100m, three-day parameters and compared them to the original 200m, six-day parameters to determine if this would improve predictive performance.

Dataset

A total number of 679 cougar clusters were used for this analysis. This resulted in 407 clusters in the training dataset, 136 clusters in the validation dataset, and 136 clusters in the testing dataset.

Table S13. AUC values from the winter cougar RFs that vary by mtry and network distance values. The cluster data used here were created with 200m, 6-day cluster parameters (which were used to generate clusters searched by field crews and reduce the likelihood of kills being missed for kill rate estimates) and 100m, 3-day parameters (reduced space-time parameters thought to increase predictive accuracy). The model with the highest AUC value was used to predict kill sites and is provided in bold text here.

100m, 3-day cluster parameters

Network	MTRY	AUC Value
200m	2	0.927
200m	3	0.926
200m	6	0.926
**400m**	**2**	**0.933**
400m	3	0.928
400m	6	0.928
600m	2	0.932
600m	3	0.927
600m	6	0.927
800m	2	0.925
800m	3	0.922
800m	6	0.922
1km	2	0.928
1km	3	0.923
1km	6	0.923

200m, 6-day cluster parameters

Network	MTRY	AUC Value
200m	2	0.891
200m	3	0.898
200m	6	0.898
400m	2	0.894
400m	3	0.894
400m	6	0.894
600m	2	0.897
600m	3	0.894
600m	6	0.894
800m	2	0.896
800m	3	0.901
800m	6	0.901
1km	2	0.907
1km	3	0.903
1km	6	0.903

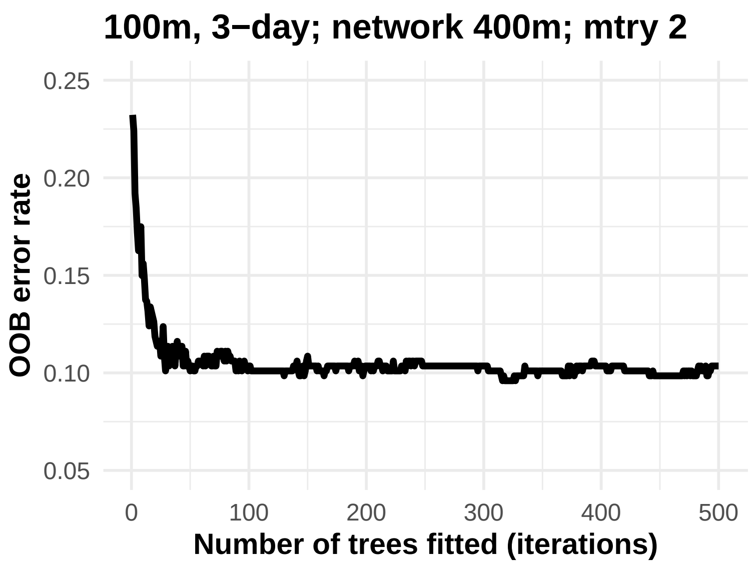

Figure S15. Out-of-bag error rates from the summer cougar RF model with the highest AUC value are plotted to look for potential overfitting as the number of trees increases.

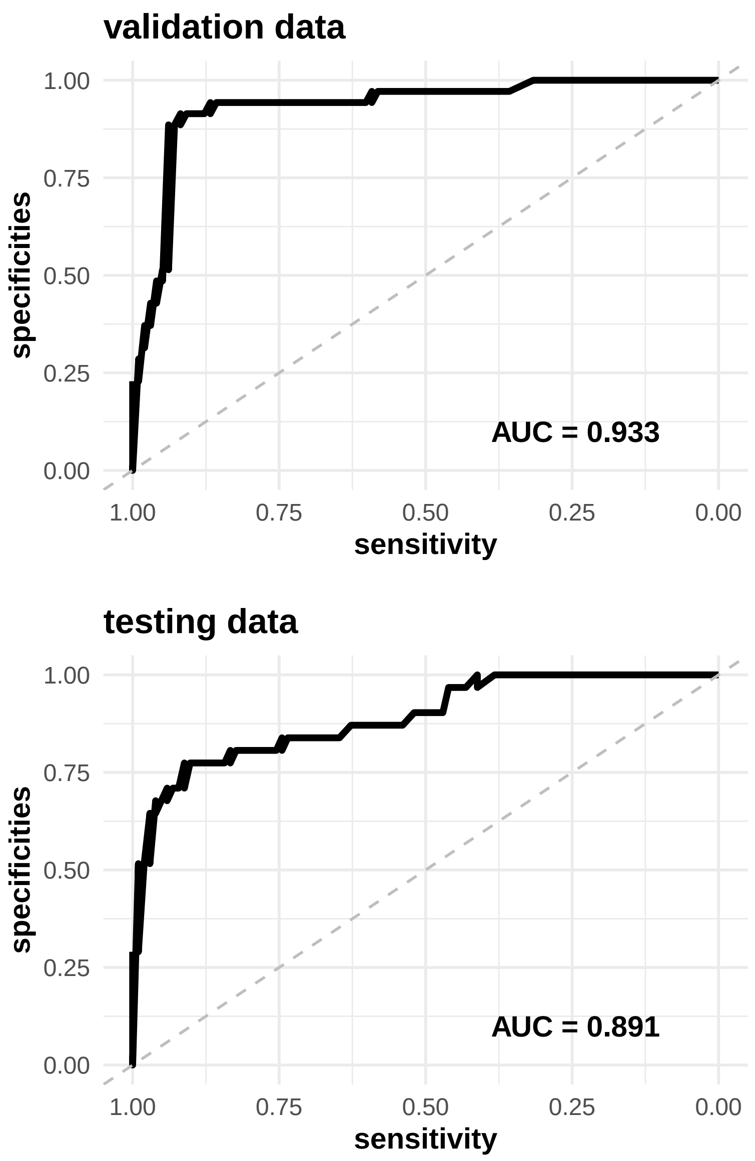

Figure S16. AUC plots from the best summer cougar model above. The top plot uses the validation data and the bottom plot uses the testing data, each of which are 20% of the cluster dataset that was not used to create the random forest model. Sensitivities (true positive rates) are plotted against specificities (true negative rates) as the threshold for assigning predictions varies.

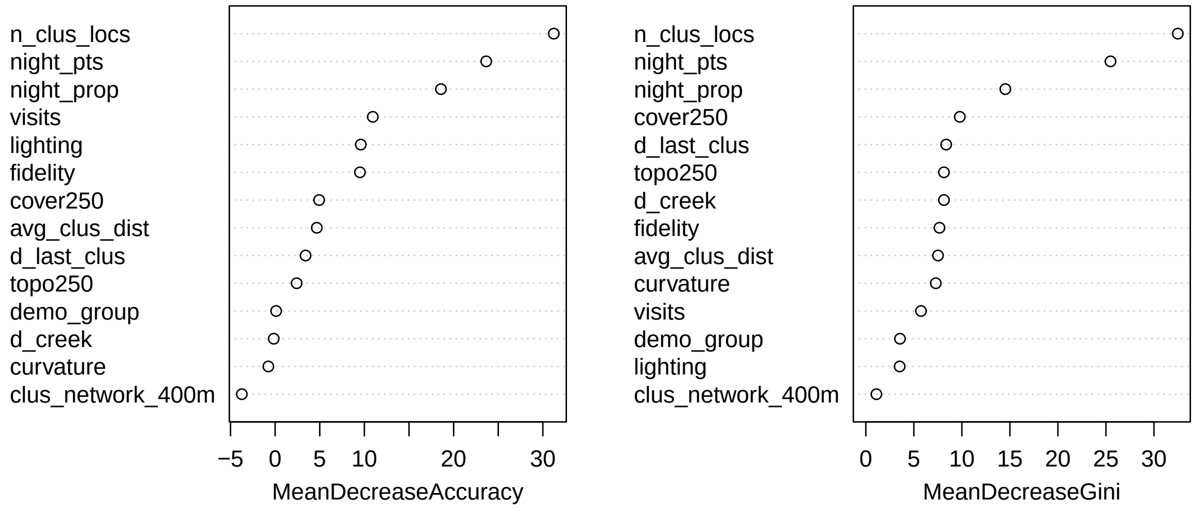

Figure S17. Variable importance plots using two metrics to assess the importance of each covariate in making predictions for the presence of kills at summer cougar clusters. First, the mean decrease in accuracy, which shows how the removal of a covariate affects the model's accuracy. Second, the mean decrease in Gini, which demonstrates how much each covariate contributes to reducing node impurity (Gini index), where higher values represent greater influence in splitting the data.

Thresholds for Classification

Here, we compare two thresholds to use for determining which clusters will be classified as kills based on the probabilities generated by the RFs. The first threshold ("maxSenSpec") is set based on the maximum value of sensitivity (true positive rate) plus specificity (true negative rate). The second threshold is set based on a specificity of 0.95 which is the lowest specificity we wanted to use in order to reduce false-positive predictions. We therefore decided to use the threshold based on the maximum sensitivity and specificity value unless the specificity for that threshold was less than 0.95.

```
## maxSenSpec sensitivity: 0.774

## maxSenSpec specificity: 0.912

## maxSenSpec threshold: 0.266

## specificity of 0.95 threshold: 0.351
```

Table S14. Confusion matrix of the summer cougar RF predictions for the testing dataset using the applicable threshold described above (here, the maxSenSpec threshold).

	Count
kill found; correct prediction	21
kill found; wrong prediction	10
no kill; correct prediction	97
no kill; wrong prediction	5

Final Model

The final model we use leverages all the cluster data along with the parameter values (cluster space and time parameters, as well as mtry and network distance) used in the model above with the highest AUC value.

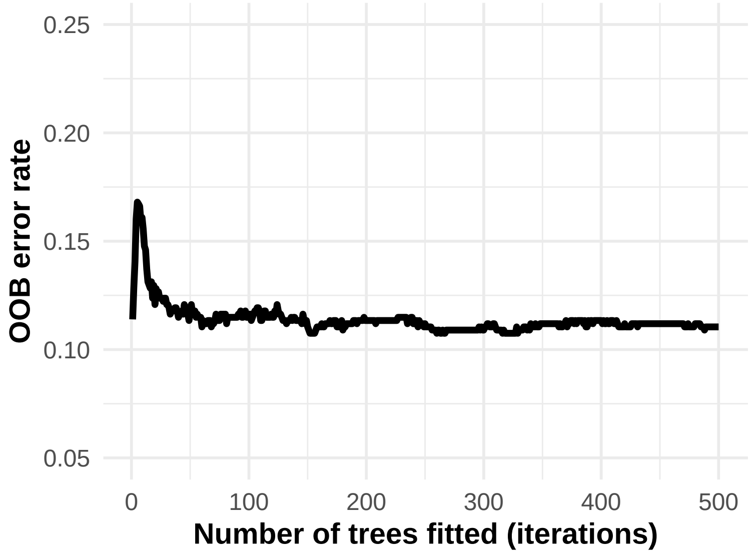

Figure S18. OOB error rates are plotted to check for potential overfitting of the final summer cougar RF model.

SI References: Appendix B

1. D.R. Cutler, T.C. Edwards, K.H. Beard, A. Cutler, K.T. Hess, J. Gibson, J.J. Lawler. Random Forests for Classification in Ecology. Ecology *88*, 2783–2792 (2007).
